# An Adverse Outcome Pathway Network for Chemically Induced Oxidative Stress Leading to (Non)genotoxic Carcinogenesis

**DOI:** 10.1021/acs.chemrestox.2c00396

**Published:** 2023-05-08

**Authors:** Christina H. J. Veltman, Jeroen L. A. Pennings, Bob van de Water, Mirjam Luijten

**Affiliations:** † Centre for Health Protection, National Institute for Public Health and the Environment (RIVM), 3720 BA Bilthoven, The Netherlands; § Division of Drug Discovery and Safety, Leiden Academic Centre for Drug Research (LACDR), Leiden University, 2333 CC Leiden, The Netherlands

## Abstract

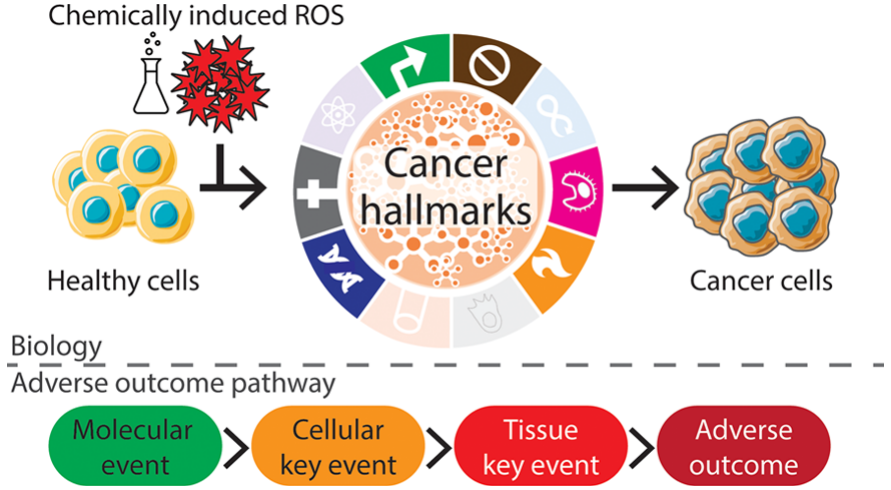

Nongenotoxic (NGTX) carcinogens induce cancer via other mechanisms than direct DNA damage. A recognized mode of action for NGTX carcinogens is induction of oxidative stress, a state in which the amount of oxidants in a cell exceeds its antioxidant capacity, leading to regenerative proliferation. Currently, carcinogenicity assessment of environmental chemicals primarily relies on genetic toxicity end points. Since NGTX carcinogens lack genotoxic potential, these chemicals may remain undetected in such evaluations. To enhance the predictivity of test strategies for carcinogenicity assessment, a shift toward mechanism-based approaches is required. Here, we present an adverse outcome pathway (AOP) network for chemically induced oxidative stress leading to (NGTX) carcinogenesis. To develop this AOP network, we first investigated the role of oxidative stress in the various cancer hallmarks. Next, possible mechanisms for chemical induction of oxidative stress and the biological effects of oxidative damage to macromolecules were considered. This resulted in an AOP network, of which associated uncertainties were explored. Ultimately, development of AOP networks relevant for carcinogenesis in humans will aid the transition to a mechanism-based, human relevant carcinogenicity assessment that involves a substantially lower number of laboratory animals.

## Introduction

Carcinogenesis is a multistep process in which normal cells transform into cancer cells by acquiring various, biologically diverse characteristics referred to as cancer hallmarks.^1−3^ At present, the International Agency for Research on Cancer (IARC) has classified 534 chemical compounds as proven human carcinogen (IARC 1), probable human carcinogen (IARC 2A) or possible human carcinogen (IARC 2B).^4^ These chemical carcinogens can induce tumor formation either through genotoxic (GTX) or nongenotoxic (NGTX) mechanisms.^5^ Compounds able to directly damage or interact with DNA are generally classified as GTX carcinogens, whereas NGTX carcinogens do not directly interact with DNA nor the cellular apparatus involved in preserving genomic integrity.^6^ Instead, NGTX carcinogens induce carcinogenesis via mechanisms including receptor activation, chronic inflammation, immune suppression, endocrine disruption, epigenetic silencing or oxidative stress.^2,7,8^ Both GTX and NGTX mechanisms of carcinogenesis eventually involve the cancer hallmarks of genomic instability, loss of proliferative control and resistance to cell death.^5^ Despite a wide spectrum of modes of action (MoAs) for NGTX carcinogens, increased cell proliferation is shown to be a fundamental key event (KE).^7,9^ Receptor activation, sustained cytotoxicity, altered signal transduction, immunosuppression and induction of oxidative stress can all contribute to stimulation of cell proliferation.^1,7^ Contrary to GTX carcinogens, NGTX carcinogens are hypothesized to induce tumorigenesis through repeated or sustained exposure resulting in prolonged perturbation or modulation of physiological processes.^10,11^

Oxidative stress leading to regenerative proliferation is a MoA relevant for carcinogenesis.^12−16^ Upon an imbalance between the generation of oxidants, such as reactive oxygen species (ROS), and their scavenging by antioxidants, oxidative stress is induced.^17^ This imbalance can arise from exposure to either endogenous or exogenous sources responsible for oxidant generation or from depletion of antioxidants.^13^ Oxidative stress is known to promote carcinogenesis through both DNA damage and impaired repair and through indirect actions influencing homeostasis and signaling.^18^ Additionally, ROS play a role in numerous stages of the multistep carcinogenic process.^19,20^

Traditionally, cancer hazard assessment requires long-term carcinogenicity studies (OECD Test Guidelines 451^21^/453^22^). There is a strong need for alternative approaches because rodent studies show limited translatability to man,^23^ raise ethical concerns, have questionable reproducibility^7,24^ and are time- and cost consuming.^23^ Currently, cancer hazard assessment predominantly relies on genetic toxicity end points, since genetic damage is considered to be key to carcinogenicity.^25^ While there is evidence of a GTX MoA for most of the substances classified by IARC, approximately 9% of these substances lack a GTX potential based on data from a battery of *in vitro* and *in vivo* genotoxicity tests,^8^ indicating that NGTX carcinogens may remain undetected in such evaluations since these act via other mechanism than direct genetic damage. Therefore, to enhance the prediction of carcinogenic potential of substances, transitioning toward a mechanism-based approach is deemed necessary.^7,25−28^ To aid this transition, adverse outcome pathways (AOPs) could serve as a framework to select appropriate new approach methodologies (NAMs) and identify research gaps.

In this article, an AOP network for chemically induced oxidative stress leading to (NGTX) carcinogenesis is proposed. We start with summarizing the main findings concerning the role of oxidative stress in the cancer hallmarks and its relation to cancer in both animals and humans. Next, possible mechanisms for chemical induction of oxidative stress are considered. The relation between oxidative stress and the cancer hallmarks is further explored by discussing the biological effects of oxidative damage to macromolecules. Ultimately, we integrate this information into an AOP network that can contribute to the development of an integrated approach to the testing and assessment (IATA) for suspected (NGTX) carcinogens inducing oxidative stress. Given the breadth of literature on oxidative stress as well as carcinogenesis, we considered it necessary to demarcate the scope of this paper. This review focuses on the role of ROS, whereas the role of reactive nitrogen species in oxidative stress and carcinogenesis is considered beyond the scope of this paper. Additionally, although we are well aware of the interrelation between oxidative stress and inflammation, we deliberately describe the discussed processes from an oxidative stress perspective.

## Methodology

A literature search was performed in October 2021 using Embase. As a search strategy (Figure S1), certain keywords such as oxidative stress/reactive oxygen species/oxygen radicals, chemical induction/carcinogen and carcinogenesis/tumorigenesis/cancer, etc. were used. After compiling a list of 539 papers, abstracts were read and papers focusing on genotoxins, effects in offspring, and antioxidants, or when the full text could not be accessed were discarded. The resulting papers were read and then either included or excluded based on redundancy of information. Some additional relevant papers were obtained through reference tracking. Papers published after October 2021 that sufficed our search strategy were read and incorporated if assessed relevant.

## Oxidative Stress in Relation to the Cancer Hallmarks

**Figure 1:**
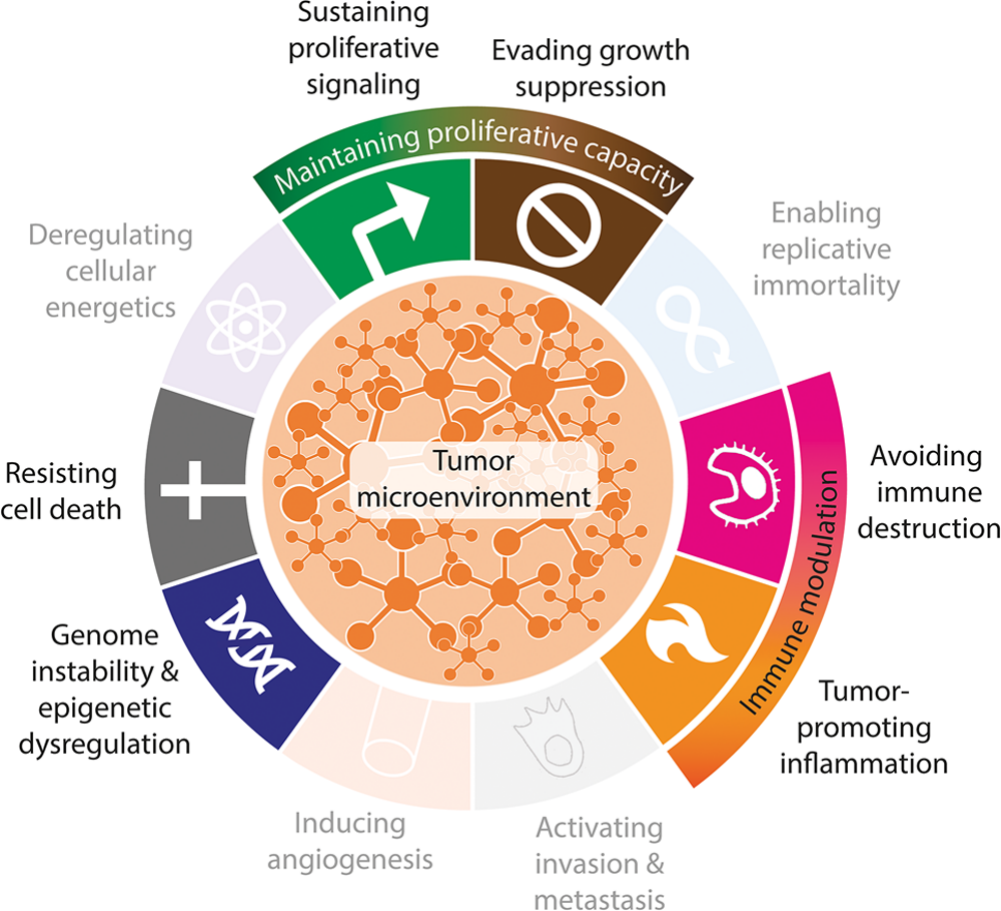
Hallmarks of cancer. The 10 hallmarks of cancer as described by Hanahan and Weinberg,^2^ supplemented with novel insights into carcinogenesis.^3,29,30^ Translucent hallmarks are not discussed in this paper. The inner circle schematically represents the signaling networks that connect the hallmarks and the tumor microenvironment.

During the process of carcinogenesis cells acquire certain characteristics referred to as cancer hallmarks.^1,2^ These hallmarks rationalize the complex biological processes involved in tumor formation.^2,29,30^ In a well-known review, Hanahan and Weinberg proposed the following six hallmarks: (1) ‘sustaining proliferative signaling’, (2) ‘evading growth suppression’, (3) ‘enabling replicative immortality’, (4) “inducing angiogenesis”, (5) “resisting cell death”, and (6) ‘activating invasion and metastasis’.^1^ Later, two emerging hallmarks and two enabling characteristics were added: ‘avoiding immune destruction’, ‘deregulating cellular energetics’, ‘genome instability and mutation’, and ‘tumor-promoting inflammation’.^2^ Since then, novel insights into carcinogenesis have led to occasional reconsideration of the cancer hallmarks^3,29,30^ (Figure 1).

A vast number of studies have described the relationship between oxidative stress and carcinogenesis.^13,16,18−20,31−33^ Generally, low concentrations of ROS are involved in regular biological processes such as transcriptional regulation, differentiation and proliferation, whereas high levels of ROS exceed the antioxidant defense system, consequently inducing oxidative stress.^34^ Carcinogenesis may occur when ROS exceed physiological levels sustainably while avoiding excessive cell death.^20^ ROS can induce tumor formation either through GTX or NGTX mechanisms.^13^ Genotoxicity occurs when ROS interact with DNA and the resulting oxidative DNA damage is not repaired prior to DNA replication. Alternatively, ROS can modulate expression of genes and proteins, such as growth factors and proto-oncogenes, which play a pivotal role in carcinogenesis, without inflicting direct DNA damage.^13,18^

Since the focus of this review is on NGTX carcinogenesis by chemical induction of oxidative stress, the cancer hallmarks ‘activating invasion and metastasis’ and ‘inducing angiogenesis’ will not be discussed. These hallmarks are considered late events in carcinogenesis, making it virtually impossible to distinguish chemically induced oxidative stress from tumor-induced oxidative stress, thereby lowering its predictive value for human health protection and relevance for chemical hazard assessment.^7^ For the cancer hallmarks ‘enabling replicative immortality’ and ‘deregulating cellular energetics’, we were unable to find sufficiently substantial, biologically relevant evidence for their relation with chemical induction of oxidative stress. Hence, the focus of this review is on the relationship between oxidative stress and the cancer hallmarks ‘maintaining proliferative capacity’ (fusion of sustaining proliferative signaling and evading growth suppression), ‘resisting cell death’, ‘immune modulation’ (merger of avoiding immune destruction and tumor-promoting inflammation), and ‘genome instability and epigenetic dysregulation’, excluding direct GTX effects.

### Maintaining Proliferative Capacity

Cancer cells are known for their ability to maintain proliferation.^2^ One mechanism through which ROS are involved in sustaining proliferative signaling is through oxidation of phosphatases and subsequent activation of signaling pathways associated with cell proliferation.^13,32,35^ To illustrate, ROS can oxidize and subsequently inhibit phosphatase and tensin homologue (PTEN), leading to activation of phosphoinositide 3 kinase (PI3K)/protein kinase B (Akt) signaling.^36,37^ Alternatively, PI3K/Akt signaling activation can be mediated by oxidation of protein tyrosine phosphatase 1B (PTP1B).^38^ Similarly, oxidation of mitogen-activated protein kinase (MAPK) phosphatase (MKP) leads to activation of MAPK/extracellular signal-regulated kinase (ERK) signaling.^39,40^ MAPK/ERK signaling can be triggered through ROS-dependent intracellular calcium release and subsequent activation of protein kinase C (PKC) as well.^41^ Notably, induction of oxidative stress can both activate and repress nuclear factor κ-light-chain-enhancer of B cells (NF-κB) signaling.^35,42^ Inhibitor of NF-κB (IκB) oxidation results in dissociation, allowing nuclear translocation of NF-κB,^43^ whereas oxidation of IκB kinase (IKK) prevents IκB degradation and subsequent NF-κB nuclear translocation.^44,45^

Alternatively, cell proliferation can be maintained through activation of activator protein 1 (AP-1). Upon oxidation of thioredoxin (TRX), apoptosis signal-regulating kinase 1 (ASK1) activity is no longer inhibited, allowing c-Jun N-terminal kinase (JNK) proteins to translocate to the nucleus for induction of AP-1.^46,47^ Activation of AP-1 induces expression of growth-stimulatory genes and suppresses cell cycle inhibitors.^13,48^ Lastly, in response to oxidative modification of Kelch like ECH associated protein 1 (KEAP1), nuclear factor erythroid 2-related factor 2 (NRF2) is activated and subsequent translocation to the nucleus leads to expression of genes involved in proliferation (e.g., Ki67 and NOTCH1).^49,50^

### Resisting Cell Death

The normal balance between proliferation and cell death is disturbed in cancer cells.^29^ ROS can disrupt this equilibrium either through inhibition of pro-apoptotic factors or through induction of antiapoptotic factors, ultimately mediating resistance to cell death. For example, ROS can activate the Akt pathway through oxidation of PTEN,^36,37^ which may lead to increased cell survival via phosphorylation and consequent inactivation of pro-apoptotic factors such as B-cell lymphoma (Bcl)-2-associated agonist of cell death (Bad), Bcl-2-associated X protein (Bax), and Bcl-2-interacting mediator of cell death (Bim).^33,51,52^ Additionally, oxidative stress can activate MAPK/p38,^53^ NF-κB^43^ and NRF2^50^ signaling, resulting in reduced caspase activity. Moreover, NRF2 activation can reduce the release of cytochrome-*c* from mitochondria, preventing apoptosis through inhibition of apoptosome formation.^54,55^ Alternatively, ROS can induce expression of antiapoptotic Bcl-2 family members by means of NRF2,^54,55^ NF-κB, and signal transducer and activator of transcription 3/5 (STAT3/5) signaling.^56^

### Immune Modulation

According to immune surveillance theory, cells are continually monitored by the body’s immune system and eliminated upon becoming cancerous.^2^ Following this principle, existing tumors have arisen from cells that somehow managed to avoid detection and elimination by the immune system. Oxidative stress can mediate avoidance of immune destruction in three ways. First, oxidative stress can induce the formation of regulatory T-cells and strengthen their immunosuppressive potency.^57^ Second, by inhibiting the interaction between the T-cell receptor and the major histocompatibility complex (MHC)-peptide complex, ROS can functionally impair cytotoxic T-cells, which play a pivotal role in immune destruction of cancer cells.^58^ Lastly, tumor-induced myeloid-derived suppressor cells (MDSCs) can inhibit T-cell proliferation in a ROS-dependent manner.^59^

Contrarily, oxidative stress is also involved in creating a tumor-promoting inflammatory microenvironment. The abundance of ROS can trigger pro-oncogenic signaling pathways, for example, NF-κB,^43^ MAPK,^40^ and STAT3,^56^ which in turn can contribute to the production of pro-inflammatory mediators such as tumor necrosis factor (TNF)-α, interleukin (IL)-1β, and IL-6.^56,60^ Consecutively, these pro-inflammatory mediators can stimulate both ROS production^61^ and pro-oncogenic signaling pathways involved in proliferation, angiogenesis, invasion, and resistance to apoptosis.^62^

### Genome Instability and Epigenetic Dysregulation

Besides direct induction of mutations as a result of oxidative DNA damage, oxidative stress can contribute to genome instability and epigenetic dysregulation. Repetitive sequences, which are particularly susceptible to DNA oxidation, can form secondary DNA structures upon oxidation.^63^ During DNA replication, recruitment of a special but error-prone DNA polymerase to these secondary DNA structures is needed for continuation of synthesis, ultimately contributing to genome instability.^63^ Furthermore, ROS-induced expression of Bcl-2, following NRF2,^54,55^ NF-κB, or STAT3/5 signaling,^56^ can inhibit DNA double-strand break (DSB) repair.^64^ Lastly, ROS-induced inflammation can cause both microsatellite and chromosomal instability via dysregulation of DNA repair enzymes, defective mitotic checkpoints, induction of DSBs, and dysregulated homologous recombination (reviewed in ref (65)).

Alternatively, ROS can cause alterations in the DNA methylation status through interaction with DNA and proteins.^15^ As a result of DNA oxidation, 8-hydroxy-2′-deoxyguanosine (8-OHdG) and 5-hydroxymethylcytosine (5hmC) may be formed.^66,67^ Consequently, DNA binding of methyl-CpG binding proteins (MBPs), epigenetic regulators responsible for DNA methyl transferase (DNMT) and histone deacetylase (HDAC) recruitment can be inhibited and these DNA regions might therefore get hypomethylated.^66,67^ Most demethylated regions are promoters belonging to oncogenes, which consequently can be activated.^15,68,69^ Additionally, methylation of repetitive and transposable elements is often lost in cancer, which can result in random integration of these elements into the genome and subsequently cause genetic instability.^15,70^ On the other hand, ROS can also mediate hypermethylation and subsequent loss of tumor suppressor promoter regions through upregulation and recruitment of DNMT and HDAC, a phenomenon frequently observed in human cancers.^15,71^ For example, hypermethylation of the human mutL homologue 1 (hMLH1) promoter region diminishes its expression,^72^ which is related to repressed activity of the mismatch repair system.^73^

## Oxidative Stress in Cancer

Oxidative stress leading to regenerative proliferation is one of the best documented mechanisms for carcinogenesis.^12−15^ About half of the IARC 1 classified substances (35 out of 86) were shown to have the ability to induce oxidative stress.^74^ Notably, chronic inflammation, associated with ROS formation and altered signaling, has become a well-recognized risk factor for various human cancers.^14,62^ Evidence for the role of oxidative stress in carcinogenesis can be found in both animals and humans.

### Animal Carcinogenicity Data

Clear evidence for oxidative stress leading to regenerative proliferation as a carcinogenic MoA in animals was derived from rodent knockout studies. Loss of antioxidant genes, such as superoxide dismutase 1 (*Sod1*),^75,76^ peroxiredoxin 1 (*Prdx1*)^77,78^ and glutathione peroxidase (*Gpx*),^79^ was shown to predispose mice to oxidative DNA damage and carcinogenesis. Moreover, contrary to wildtype mice, mice with a knockout of cytochrome P450 2E1 (*Cyp2e1*; a known electron leaker during xenobiotic metabolism) were shown to not manifest hepatotoxicity nor regenerative proliferation upon exposure to chloroform.^80^ In another study, *Cyp2e1*-null mice showed no oxidative damage phenotype whereas wildtype and humanized *Cyp2e1* mice did.^81^ Furthermore, impaired liver regeneration has been observed in rodents lacking either AP-1 (c-Jun monomer) or NF-κB, both are essential transcription factors for hepatic regenerative proliferation.^82,83^ Inhibition of IL-1α^84^ or hematopoietic depletion of inhibitor of NF-κB kinase β (*Ikkβ*)^85^ in mice resulted in decreased regenerative proliferation. Lastly, *Jnk1* knockout mice showed impaired cellular proliferation and subsequent decreased liver carcinogenesis.^86^

In a project by the European Partnership for Alternative Approaches to animal testing (EPAA), 170 NGTX carcinogenic agrochemicals were evaluated, with the aim to assess the tumor types induced and organs affected and to identify the various MoAs underlying the carcinogenic potential.^10^ Mechanistic information collected on the NGTX carcinogens resulted in a list of nine MoAs, including sustained cytotoxicity leading to regenerative proliferation. Of the 96 substances for which a MoA could be established, 49 (51%) were presumed to induce sustained cytotoxicity. This MoA has been observed for tumors in the liver, kidney, stomach, bladder, and intestine.^10^ Induction of oxidative stress is a central event within the MoA sustained cytotoxicity.^87^ However, only for four of the 49 substances, induction of oxidative stress was explicitly mentioned.^10^ Together these four substances induced seven unique treatment-related tumors affecting the liver, spleen and lymphoid system.^10^

### Human Carcinogenicity Data

Evidence for the role of oxidative stress in carcinogenesis in humans mainly comes from epidemiological studies. Increased risk of cancer of the liver, kidney, blood, immune system, bladder and gastrointestinal tract has been reported in humans after exposure to carcinogens known to operate via oxidative stress in animals.^88−96^ For instance, GTX carcinogens (pharmaceuticals) that induce oxidative DNA damage, such as cyclophosphamide, etoposide, tamoxifen and azathioprine,^97,98^ have epidemiologically been linked to elevated cancer risk.^89^ Epidemiological evidence for NGTX carcinogens operating via oxidative stress exist as well; a few examples are described here. In a large cohort study of 23,829 sawmill workers in British Columbia, substantial evidence of an association between pentachlorophenol exposure and the incidence of liver cancer, non-Hodgkin lymphoma and multiple myeloma was found.^90^ Additionally, occupational exposure to pentachlorophenol might elevate the risk of hematopoietic,^99^ neurological and digestive tract cancer.^100^ Evidence for oxidative stress induction following pentachlorophenol exposure has been found both in cells with a human origin and in mice.^101,102^ Another example is exposure to trichloroethylene, for which an epidemiological link to increased risk of kidney cancer,^88,92−95^ non-Hodgkin lymphoma,^92,94,103^ and liver cancer^94,96^ has been found. Trichloroethylene has been shown to induce oxidative stress in a human hepatic cell line^104^ and in rats.^105^ Moreover, in a prospective cohort of pesticide applicators, an association between thyroid cancer and lindane exposure was observed.^106^ Agricultural use of lindane also has been associated with an elevated risk of non-Hodgkin lymphoma.^107,108^ In rats, lindane exposure was shown to induce oxidative stress.^109^

Another line of evidence for human relevance derives from genetic variation in oxidative-stress related genes and cancer susceptibility in the human population.^18^ Single nucleotide polymorphisms (SNPs) in antioxidant enzymes SOD and catalase (CAT) have been linked to increased cancer incidence and susceptibility.^110^ Moreover, SNPs altering the function of AP-endonuclease 1 (APE1) and 8-oxo-guanine DNA glycosylase (OGG1), DNA repair genes primarily involved in base excision repair of oxidative DNA damage,^18^ have been linked to increased cancer risk in humans.^111−113^

Next to this, biomarkers of oxidative stress can be found in cancer patients as well, though it should be noted that these are not indicative of a role for oxidative stress in tumor initiation. Increased total oxidant, decreased total antioxidant,^114−119^ elevated malondialdehyde (MDA)^114,116,120,121^ and increased protein carbonyl serum levels^117−119,122^ are found in cancer patients compared to healthy controls. Moreover, the catalogue of somatic mutations in cancer (COSMIC) has identified a mutational signature (SBS18) in human cancers which is associated with oxidative DNA damage.^123^ Finally, 8-OHdG, a biomarker for oxidative DNA damage, can be quantified in biopsies and urinary samples from cancer patients. 8-OHdG levels were found to be significantly increased in chronic hepatitis C patients with hepatocellular carcinoma (HCC) compared to chronic hepatitis C patients without HCC.^124^ Both 8-OHdG and NRF2 expressions were found to be significantly elevated in cancerous tissue compared to noncancerous tissue of HCC patients.^125^ Again, these biomarkers are indicative of oxidative stress in the cancer patient which is more likely the consequence rather than the cause of the tumor.

## Chemical Induction of Oxidative Stress

Chemical substances can induce oxidative stress through direct or indirect mechanisms. Direct mechanisms include mitochondrial and extramitochondrial production of oxygen radicals, whereas impact on the antioxidant defense system is considered an indirect mechanism.^13^ Notably, chemical induction of oxidative stress is not restricted to carcinogens nor carcinogenesis.^126−128^

### Formation of Reactive Oxygen Species

An example of a direct mechanism of oxidative stress induction is mitochondrial production of oxygen radicals. Interaction of substances with mitochondria, especially mitochondrial complex I and III,^13^ during oxidative phosphorylation is considered to be central in the formation of ROS.^129^ By blocking electron transport, a substance can induce mitochondrial membrane depolarization, and consequently ROS production.^130^

Another mechanism of ROS formation is closely related to the biotransformation of a substance. During detoxification of substances, CYP enzymes are induced.^14^ CYP enzymes catalyze the transfer of oxygen to the substrate, a process thoroughly associated with nicotinamide adenine dinucleotide phosphate (NADPH) oxidase.^48^ Upon disturbance of this association, electrons derived from NADPH can reduce CYP-oxygen complexes, consequently generating ROS.^14,18,48^ Moreover, various CYP enzymes, specifically CYP2E1, can leak electrons during the course of their catalytic cycle, producing ROS in the process.^48,131,132^ CYP1A, CYP1B1, CYP1D1 and CYP3A4 have also been described to produce ROS during their catalytic cycle,^133^ either through direct interaction with a substance or through activation of the aryl hydrocarbon receptor (AhR) in case of CYP1A1.^134^

### Impacting the Antioxidant Capacity

An indirect mechanism of oxidative stress induction is reduction of antioxidants, of which the primary function is to protect the body against overload of oxidants.^135^ The antioxidant defense system consists of three parts: enzymes, vitamins and minerals.^135,136^ This review focuses on enzymatic antioxidants since we consider these most relevant for exposure to environmental chemicals. The main human enzymatic antioxidants include SOD, CAT, GPX and PRDX.^135,137^ SOD converts superoxide radicals to hydrogen peroxide.^138^ Next, hydrogen peroxide can be converted by either CAT, GPX or PRDX.^137^ CAT directly converts hydrogen peroxide in water and oxygen,^139^ whereas GPX and PRDX require cofactors such as reduced glutathione (GSH) or reduced TRX, respectively, to convert hydrogen peroxide in water while oxidizing GSH and TRX.^137,140^ Glutathione reductase (GR) and thioredoxin reductase (TR) can convert oxidized GSH and TRX, respectively, back to their reduced states by converting NADPH to NADP^+^.^137,140^ Glutathione-*S*-transferase (GST) uses GSH for oxidant detoxification as well.^137^

## Oxidative Damage to Macromolecules

ROS can interact with and damage macromolecules such as lipids, proteins and DNA.^19^ Through interaction with these macromolecules, ROS can affect multiple cellular processes such as proliferation, inflammation and cell death.^31^ Notably, different types of ROS can have distinct effects on macromolecules. For example, hydrogen peroxide mostly oxidizes cysteine residues of proteins reversibly, controlling protein activity similarly to other post-translation modifications (e.g., phosphorylation), whereas highly reactive ROS, such as superoxide and hydroxyl radicals, are most likely to induce lipid peroxidation and cell death.^137^

### Oxidative Damage to Lipids

Lipid peroxidation is the process in which ROS break down polyunsaturated fatty acids, damaging lipid-containing cellular components, such as the cell membrane, mitochondria, and the endoplasmic reticulum (ER).^141−143^ Membrane permeability and fluidity and activity of membrane-bound proteins can be affected by lipid peroxidation.^129,144^ Importantly, products of lipid peroxidation, such as MDA and 4-hydroxynonenal (4-HNE),^141,142^ can react with other macromolecules further impairing cellular function.^143,145^

**Figure 2:**
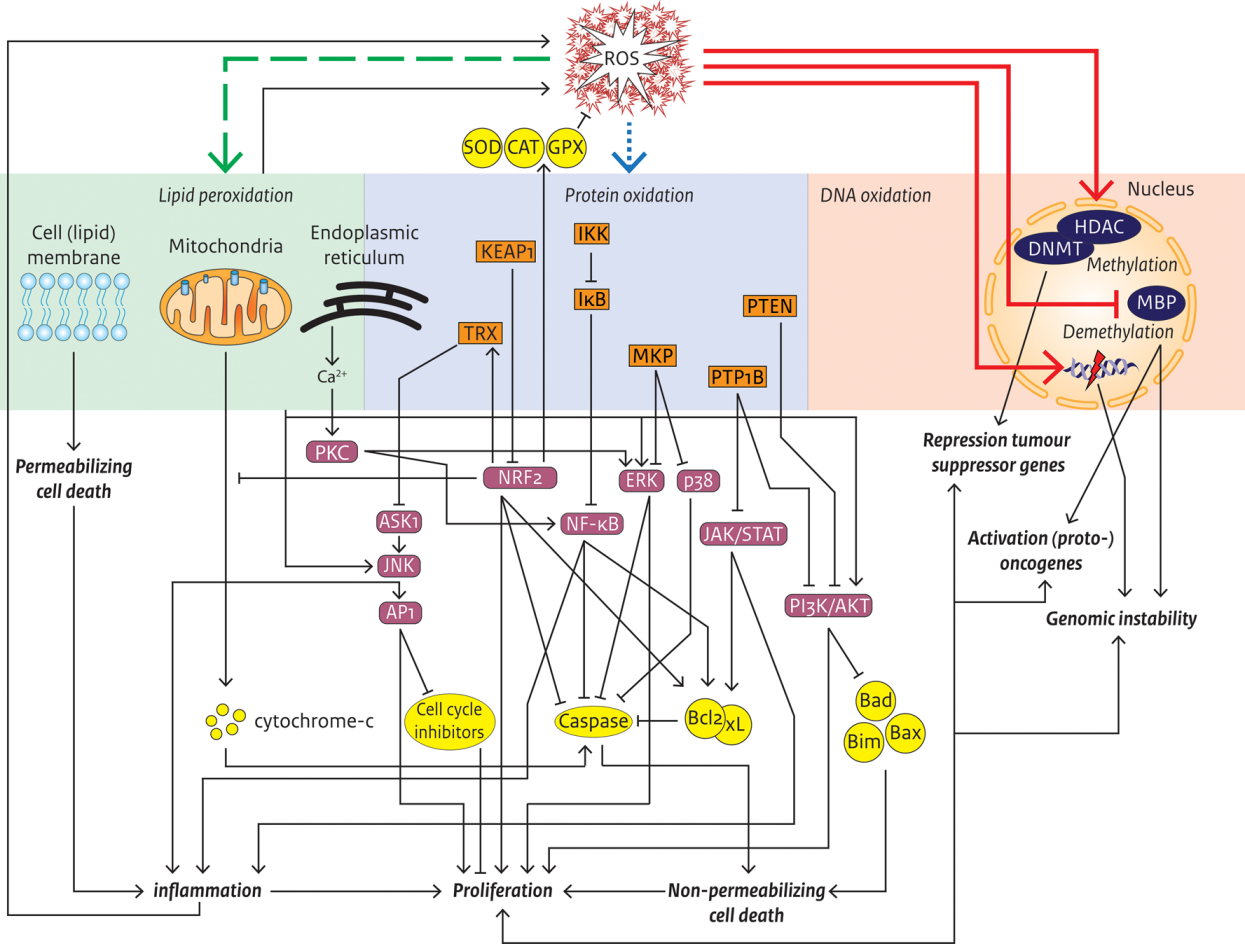
Schematic representation of ROS-induced damage and biological effects in relation to carcinogenesis. ROS can interact with and damage lipids (green/left), proteins (blue/middle) and DNA (red/right). Through signaling pathways, oxidative modification of macromolecules can contribute to various cancer hallmarks and, ultimately, carcinogenesis. Inflammation also affects neighboring cells. First layer effector molecules are depicted in orange/rectangles, second layer effector molecules in purple/rounded rectangles, and third layer effector molecules in yellow/circles. ROS: reactive oxygen species; SOD: superoxide dismutase; CAT: catalase; GPX: glutathione peroxidase; PKC: protein kinase; NF-κB: factor κ-light-chain-enhancer of B cells inhibitor; IκB: Inhibitor of NF-κB; IKK: IκB kinase; PTEN: phosphatase and tensin homologue; PI3K: phosphoinositide 3 kinase; AKT: protein kinase B; PTP1B: protein tyrosine phosphatase 1B; JAK/STAT: Janus kinase/signal transducer and activator of transcription protein; MKP: MAPK phosphatase; ERK: extracellular signal-regulated kinase; TRX: thioredoxin; AKS1: apoptosis signal-regulating kinase 1; JNK: c-Jun N-terminal kinase; AP1: activator protein 1; KEAP1: Kelch like ECH associated protein 1; NRF2: nuclear factor erythroid 2-related factor 2; HDAC: histone deacetylase; DNMT: DNA methyltransferase; MBP: methyl binding protein; Bcl2: B-cell lymphoma 2; Bcl-xL: B-cell lymphoma extra-large; Bad: Bcl-2-associated agonist of cell death; Bim: Bcl-2-interacting mediator of cell death; Bax: Bcl-2-associated X protein.

Lipid peroxidation can affect cell signaling pathways involved in proliferation, inflammation and cell death (Figure 2). It is important to note that, similar to ROS itself, low levels of lipid peroxidation have proliferative effects, whereas high levels induce cell death.^146^ For example, low concentrations of 4-HNE can trigger activation of PI3K/Akt and MAPK/ERK signaling pathways, while high concentrations of 4-HNE are cytotoxic.^146^ Lipid peroxidation mediated cytotoxicity can either lead to cell death or uncontrolled cell growth.^147,148^ Moreover, lipid peroxidation products can induce inflammatory responses through JNK.^149^

### Oxidative Modification of Proteins

Oxidative damage can reduce, increase, modify or completely abrogate biological activity of proteins.^143^ Examples of oxidized proteins are protein carbonyl derivates, advanced oxidation protein products, and advanced glycation end products.^141^ During oxidative stress, proteins can be temporarily oxidized leading to altered protein function and signaling.^150^ Oxidized receptor proteins can modify the transfer of signals^13^ or enzymatic activity may be reduced due to oxidative alteration.^36−40,43^ One important example is ROS-mediated cysteine modification of KEAP1, resulting in nuclear translocation and transcriptional activation of NRF2.^50^ Alternatively, protein oxidation can also be irreversible, such as protein carbonylation, resulting in loss of function due to protein aggregation and degradation.^150^ In addition, accumulation of oxidized proteins may result in impaired cellular function and apoptosis.^143^ Furthermore, oxidative stress can lead to misfolding of proteins in the ER, consequently inducing ER stress.^151^

Oxidative damage to proteins can affect cell proliferation, survival and inflammation (Figure 2). Upon oxidation of TRX^46^ or phosphatases,^36−40,43^ such as MKP, PTEN, PTP1B, and IκB, proliferative signaling is stimulated. Alternatively, oxidative stress can result in intracellular release of calcium,^152^ consequently stimulating PKC signaling, which in turn can activate MAPK/ERK and NF-κB signaling pathways.^41^ Moreover, protein oxidation can enhance survival through oxidation of PTEN, followed by PI3K/Akt activation and subsequent inactivation of pro-apoptotic Bad, Bax, and Bim.^33,51,52^ Additionally, oxidation of IκB lifts its inhibitory effect on NF-κB signaling, which can both reduce caspase activity^43^ and induce expression of antiapoptotic Bcl-2 family members.^56^ This can also be mediated by oxidation of KEAP1 and subsequent activation of NRF2 signaling.^50,54,55^ Lastly, NF-κB signaling contributes to inflammation via the production of pro-inflammatory mediators.^56,60^

### Oxidative Damage to DNA

ROS-induced oxidative DNA modification may result in alteration of the DNA methylation status^15^ or genomic instability following DNA oxidation-induced secondary structures.^63^ These alterations in DNA methylation, resulting from oxidative modification of MBPs, DNMTs, and HDACs,^66,67^ can activate proto-oncogenes, such as c-Myc, Kras and c-Jun, as a result of promoter hypomethylation,^68,69^ and inactivate tumor suppressors, for instance RUNX3 (RUNX family transcription factor 3) and hMLH1, following promoter hypermethylation^72,153^ (Figure 2). Altered regulation of proto-oncogenes and tumor suppressor genes is pivotal in carcinogenesis.^154^

### Relation with the Cancer Hallmarks and Carcinogenesis

Oxidative damage to lipids, proteins and DNA can affect multiple cancer hallmarks, including maintaining proliferative capacity, immune modulation, resistance to cell death, and genomic instability and epigenetic dysregulation (Figure 2). Ultimately, through contribution to these cancer characteristics, carcinogenesis can be initiated and promoted.^1,2^ Notably, cancer hallmarks can be induced through oxidative damage to one specific class of macromolecules or to multiple (Figure 2). Importantly, oxidative damage mediated cell death can be compensated by regenerative proliferation.^155,156^

## Proposed AOP Network for Chemical Induction of Oxidative Stress Leading to (Non)genotoxic Carcinogenesis

**Figure 3:**
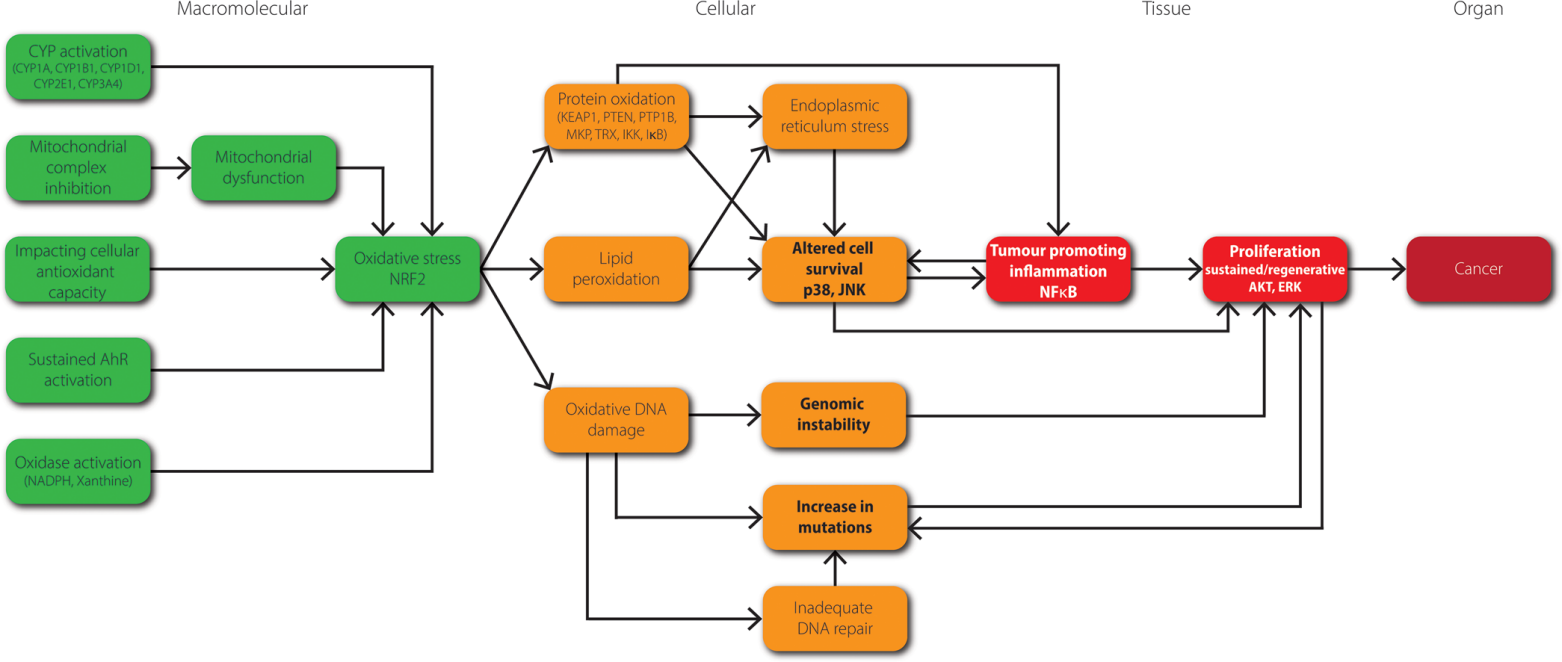
Proposed network of AOPs for chemically induced oxidative stress leading to carcinogenesis. Chronic or prolonged activation of the molecular initiating event and subsequent key events is required for this AOP network to trigger the adverse outcome. Molecular initiating events are depicted in green, cellular effects in orange, tissue effects in bright red and the adverse outcome in dark red. Cancer hallmarks are depicted in bold. Italicized text represent associated signaling pathways. Examples of cytochrome P450 enzymes, oxidases and key proteins in protein oxidation are given in parentheses.

Through structuring the above-described information regarding the role of oxidative stress in the cancer hallmarks, chemical induction of oxidative stress and oxidative damage to macromolecules, we have developed an AOP network for chemically induced oxidative stress leading to carcinogenesis (Figure 3). Notably, this is an outline AOP network that requires further development especially a detailed description of the KEs and key event relationships (KERs). A preliminary weight of evidence (WoE) evaluation was performed (Table S1A–D) according to OECD guidance.^157^ Adjacent KERs were assessed for both biological plausibility (Table S1A) and empirical evidence including consideration of contradictions (Table S1C), while KEs were assessed for essentiality (Table S1B). For this, we relied on the WoE assessments available in the AOP-Wiki (aopwiki.org) as well as scientific literature. Details are given in Tables S1A–D, with Table S1D providing a summary of the WoE for each of the adjacent KERs. Overall, the biological plausibility for the direct relationships in this AOP network is fairly strong. Evidence from empirical studies partly supports this assessment, but for some KERs the number of published studies appeared to be limited. The essentiality of the KEs in the AOP network, which can be demonstrated by modulating one KE and observing concordance in the downstream KEs, was considered as moderate. Based on this, we consider the overall WoE of the AOP network moderate to strong: certain branches are very data rich, providing strong evidence, whereas other branches require more data to be generated. Additionally, to further examine this AOP network, a noncomprehensive list of possible assays for KEs and reference chemicals for these assays is provided in Table S2.

The AOP network consists of five molecular initiating events (MIEs), namely activation of specific CYPs, inhibition of mitochondrial complexes, impacting on cellular antioxidant capacity, sustained AhR activation, and oxidase activation, which may all lead to oxidative stress. Following the central KE oxidative stress, three parallel KEs can be induced: lipid peroxidation, protein oxidation and oxidative DNA damage. Sufficient activation of these KEs can result in ER stress, altered cell survival, and/or genomic instability. Altered cell survival can result in tumor promoting inflammation, which in turn can lead to sustained or regenerative proliferation. Moreover, both genomic instability and altered cell survival can also result in regenerative or sustained proliferation. Sufficient and persistent activation of this KE can lead to tumor formation. Notably, both sustained or regenerative proliferation and mutations are pivotal in carcinogenesis, and their relationships to carcinogenesis are interlaced. Importantly, chronic or prolonged activation of the MIEs and subsequent KEs is necessary to trigger cancer.

## Discussion and Conclusion

In the preceding sections, we reported evidence for the role of oxidative stress in carcinogenesis along the lines of the cancer hallmarks. We summarized the main findings concerning the role of oxidative stress in cancer in both animals and humans. Lastly, we described how chemicals can induce oxidative stress, and how oxidative stress to macromolecules relates to cell death, ER stress, inflammation, proliferation, and genomic instability. Our efforts have led to the development of an AOP network for chemically induced oxidative stress leading to carcinogenesis (Figure 3), which can be used to guide the development of an IATA for suspected NGTX carcinogens inducing oxidative stress. Notably, many other mechanism relevant for tumorigenesis exist. These were considered beyond the scope of this review and are therefore not included in the presented AOP network.

Within the AOP-Wiki,^158^ 53 AOPs mentioning either oxidative stress or ROS as KE exist at the time of writing this review (Table S3). Of the 53 AOPs, only 12 cover cancer as an adverse outcome (AO), highlighting the diverse role of oxidative stress. In these 12 AOPs, various organs are linked to cancer resulting from induction of oxidative stress, including the liver, lung, breast, stomach and mesothelium. The diversity in target sites for the AO has implications for hazard assessment since quantitative differences between organs presumably affect the point of departure. Additionally, several currently existing AOPs link cytotoxicity to carcinogenesis (Table S4). Although these AOPs do not specifically mention induction of oxidative stress, this is known to be closely linked to cytotoxicity.^87^ Notably, chronic or sustained activation of KEs is necessary for carcinogenesis to occur.

The described AOP network is exploratory and requires further development to elucidate the associated uncertainties. One uncertainty relates to the relationship between mitochondrial dysfunction and oxidative stress. While some AOPs suggest that oxidative stress is the consequence of mitochondrial dysfunction, others suggest that oxidative stress is the cause. Here it should be noted that AOPs are a simplified representation of complex biological networks. The discrepancy between different AOPs is most likely the result of this simplification. We chose to depict mitochondrial dysfunction as the causative factor for oxidative stress since there is evidence for chemical interference with mitochondrial complexes leading to oxidative stress.^130^ However, ROS have been described to disrupt mitochondrial function, and this should be taken into account upon quantification of the KER. Another feedback loop within the AOP network includes damage to macromolecules by lipid peroxidation products,^145^ causing reinforcement of the effect of oxidative stress. Moreover, inflammation can both be portrayed as the causative and consequential factor in relation to oxidative stress. This likely results from the interrelation between oxidative stress and inflammation as an abundance of ROS can trigger production of pro-inflammatory cytokines, which in turn can stimulate ROS formation, resulting in a positive feedback loop.^62^ Since the focus of our AOP network is chemically induced oxidative stress, we depict oxidative stress as the cause and inflammation as the consequence. Similarly, oxidative stress can induce ER stress through protein oxidation, and ER stress can, in turn, cause oxidative stress.^151^ Lastly, altered cell survival and inflammation appear to be intertwined KEs. Cell death is known to trigger inflammation through the production of damage-associated molecular patterns (DAMPs),^159^ and inflammation can induce cell death via a process referred to as pyroptosis (reviewed in^160,161^). Importantly, all biologically relevant feedback loops should be taken into account when quantifying the AOP network.

We would like to give a few considerations concerning the application of the proposed AOP network for *in vitro* testing. First, both the macromolecular and cellular KEs can be measured *in vitro* using a relatively simple model system, for example, HepG2 cells. Despite quantitative uncertainties due to the cancer origin, such cell lines can be used for high-throughput screening after which more complex and relevant model systems can be used for further testing. One important challenge here is the definition of oxidative stress. At present, no threshold to discern physiological ROS levels from pathological ROS levels exist. Second, since prolonged activation of KEs is required for carcinogenesis to occur, repeated, low-dose exposure is hypothesized to be more relevant compared to single, high-dose exposure. *In vitro* assays are still limited in their duration and applicability for testing repeated-dose toxicity. Possibly, recovery experiments can shed light on the sustainability of induced effects. Lastly, no regulatory accepted *in vitro* assays for measuring proliferation currently exist. Additionally, the AO cannot be measured *in vitro*. Therefore, other downstream KEs such as altered cell survival and tumor promoting inflammation are thought to be key in distinguishing carcinogens from noncarcinogens. *In vitro* testing of the KE tumor promoting inflammation requires a complex model system containing multiple cell types, e.g., hepatocytes, Kupffer cells and stellate cells. For the KE altered cell survival interpretation at the moment in unclear. Both an increase and a decrease in cell survival can be linked to carcinogenesis, for the latter in combination with regenerative proliferation. Future research will have to elucidate how both different types of cell death and quantitative differences in cell survival are to be interpreted in relation to carcinogenesis.

For regulatory application of this AOP network, quantification of the various KERs is essential. Not all substances that induce oxidative stress through one of the proposed MIEs are known to cause cancer. For example, although acetaminophen (MIE: CYP2E1 activation)^162^ and diclofenac (MIE: mitochondrial dysfunction)^163^ are hepatotoxicants that are able to induce oxidative stress, they are not known as (human) carcinogens. We hypothesize that quantitative differences in certain KERs, specifically those associated with the cancer hallmarks, are central in this distinction of carcinogenic potential. To illustrate, acetaminophen is highly hepatotoxic and possibly does not cause liver cancer because death by liver failure will precede carcinogenesis.^131^ Next to this, toxicokinetic differences between substances are presumed to affect this inequity in AO induction as well. Furthermore, target sites can differ in their antioxidant capacity and varying responses to oxidative stress by the microenvironment are thought to influence carcinogenesis.^137^ Lastly, increased cellular proliferation does not exclusively lead to carcinogenesis, healthy regeneration can occur as well. Therefore, sustained perturbation of signaling, ultimately impairing normal regeneration and homeostasis, is expected to be fundamental in malignant transformation. Hazard assessment will thus have to qualitatively and quantitatively cover the KERs in the proposed AOP network and consider tissue-specific differences in sensitivity.

## Supporting Information Available

Figure S1: Flowchart search strategy. Table S1: Weight of evidence assessment of the AOP network, conducted according to OECD guidance. Table S2: Possible assays for the AOP network and reference chemicals for these assays Table S3: AOPs from the AOP-wiki with oxidative stress or reactive oxygen species as key event. Table S4: AOPs from the AOP-wiki with cytotoxicity as key event and cancer as adverse outcome not mentioning oxidative stress or reactive oxygen species as key event (PDF)

## Abbreviations

4-HNE: 4-hydroxynonenal
5hmC: 5-hydroxymethylcytosine
8-OHdG: 8-hydroxy-2′-deoxyguanosine
AhR: aryl hydrocarbon receptor
Akt: protein kinase B
AO: adverse outcome
AOP: adverse outcome pathway
AP-1: activator protein 1
APE1: AP-endonuclease 1
AKS1: apoptosis signal-regulating kinase 1
Bad: Bcl-2-associated agonist of cell death
Bax: Bcl-2-associated X protein
Bcl-xL: B-cell lymphoma extra-large
Bim: Bcl-2-interacting mediator of cell death
CAT: catalase
CYP: cytochrome P450
DAMP: damage-associated molecular pattern
DNMT: DNA methyl transferase
DSB: double-strand break
EPAA: European partnership in alternative approaches to animal testing
ER: endoplasmic reticulum
ERK: extracellular signal-regulated kinase
GPX: glutathione peroxidase
GR: glutathione reductase
GSH: glutathione
GST: glutathione-*S*-transferase
GTX: genotoxic
HCC: hepatocellular carcinoma
HDAC: histone deacetylase
hMLH1: human mutL homologue 1
IARC: International Agency for Research on Cancer
IATA: integrated approach to testing and assessment
IκB: inhibitor of NF-κB
IKK: IκB kinase
IL: interleukin
JAK: Janus kinase
JNK: c-Jun N-terminal kinase
KE: key event
KEAP1: Kelch-like ECH associated protein 1
KER: key event relationship
MAPK: mitogen-activated protein kinase
MBP: methyl binding protein
MDA: malondialdehyde
MDSC: myeloid-derived suppressor cell
MHC: major histocompatibility complex
MIE: molecular initiating event
MKP: MAPK phosphatase
MoA: mode of action
NADPH: nicotinamide adenine dinucleotide phosphate
NAM: new approach methodology
NF-κB: factor κ-light-chain-enhancer of B cells inhibitor
NGTX: nongenotoxic
NRF2: nuclear factor erythroid 2-related factor 2
OGG1: 8-oxo-guanine DNA glycosylase
PI3K: phosphoinositide 3 kinase
PKC: protein kinase
PRDX: peroxiredoxin
PTEN: phosphatase and tensin homologue
PTP1B: protein tyrosine phosphatase 1B
ROS: reactive oxygen species
RUNX3: RUNX family transcription factor 3
SNP: single nucleotide polymorphism
SOD: superoxide dismutase
STAT: signal transducer and activator of transcription protein
TNF: tumor necrosis factor
TR: thioredoxin reductase
TRX: thioredoxin
WoE: weight of evidence
