# Supplementary material for: An Adverse Outcome Pathway Network for Chemically Induced Oxidative Stress Leading to (Non)genotoxic Carcinogenesis
Source: Chem Res Toxicol. 2023 May 8;36(6):805–17. doi: 10.1021/acs.chemrestox.2c00396 (PMC10283045; doi:10.1021/acs.chemrestox.2c00396)
Supplement: Supplementary file 1 — tx2c00396_si_001.pdf [file tx2c00396_si_001.pdf]

Supporting Information

An Adverse Outcome Pathway Network for Chemically Induced Oxidative Stress Leading to (Non)genotoxic Carcinogenesis

Christina H. J. Veltman[†§]*, *Jeroen L. A. Pennings*[†], *Bob van de Water*[§], *Mirjam Luijten*[†]

[†]Centre for Health Protection, National Institute for Public Health and the Environment (RIVM), Bilthoven, The Netherlands. [§]Division of Drug Discovery and Safety, Leiden Academic Centre for Drug Research (LACDR), Leiden University, Leiden, The Netherlands.

AUTHOR INFORMATION

Corresponding Author

*Christina H. J. Veltman, National Institute for Public Health and the Environment (RIVM), P.O. Box 1, 3720 BA Bilthoven, The Netherlands, kirsten.veltman@rivm.nl.

TABLE OF CONTENTS

Figure S1. Flowchart search strategy.	S2
Table S1. Weight of evidence assessment of the AOP network, conducted according to OECD guidance.	S3
Table S2. Possible assays for the AOP network and reference chemicals for these assays.	S13
Table S3. AOPs from the AOP-wiki with oxidative stress or reactive oxygen species (ROS) as key event.	S15
Table S4. AOPs from the AOP-wiki with cytotoxicity as key event and cancer as adverse outcome not mentioning oxidative stress or reactive oxygen species (ROS) as key event.	S18
References	S19

Figure S1. Flowchart search strategy. The left column depicts search terms used for inclusion of papers, the right column depicts terms, topics and considerations used for exclusion of papers. Exclusion was performed manually after reading of abstracts. Searches were performed in October 2021 using Embase. Papers published after this date that did suffice the search criteria were manually included. Some additional relevant papers that were encountered through reference tracking were included as well. Redundant papers were not included in the final manuscript.

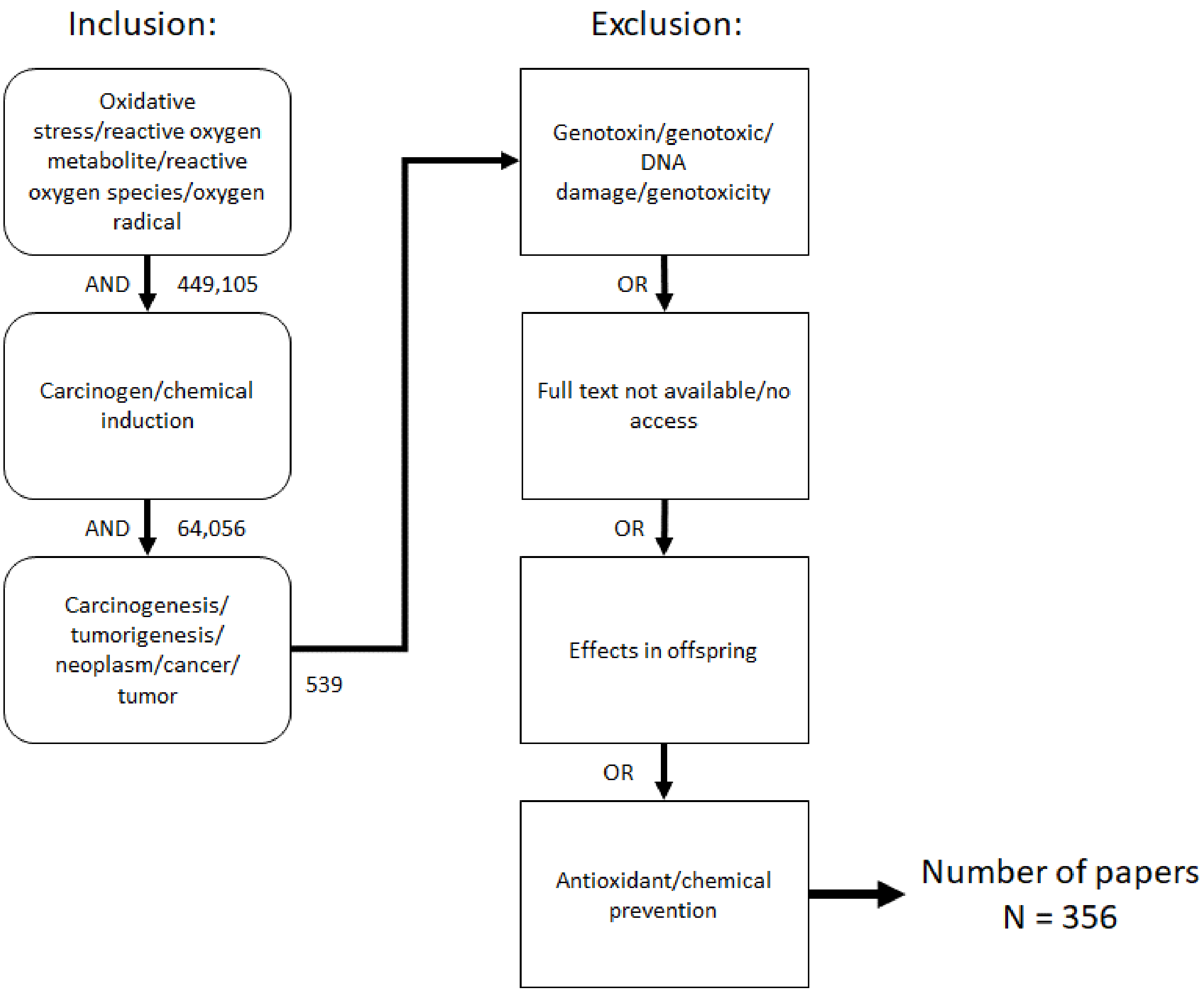

Table S1. Weight of evidence assessment of the AOP network, conducted according to OECD guidance. A) Biological plausibility of adjacent key event relationships; B) Essentiality of key events; C) Empirical support for adjacent key event relationships; D) Overall assessment of adjacent key event relationships.

**A) Biological plausibility of adjacent key event relationships**			
**KER**	**Level of support**	**Mechanism**	**AOP wiki**
CYP activation (CYP1A, CYP1B1, CYP1D1, CYP2E1, CYP3A4) leading to oxidative stress	Moderate	Specifically CYP2E1 is a known leaker of reactive oxygen species. Other CYPs (1A, 1B1, 1D1 and 3A4) can also produce ROS during their catalytic cycle.[1-4]	KER#1512
Mitochondrial complex inhibition leading to mitochondrial dysfunction	Strong	It is broadly accepted that mitochondrial complex inhibition results in decreased oxidative phosphorylation.[5]	KER#1781, KER#1782, KER#1783
Mitochondrial dysfunction leading to oxidative stress	Moderate	Mitochondria and oxidative phosphorylation are thought to be central in the formation of ROS.[6]	
Impacting cellular antioxidant capacity leading to oxidative stress	Moderate	Antioxidant enzymes play an important role in protection against oxidants.[7-12]	AOP#413
Sustained AhR activation leading to oxidative stress	Weak	AhR activation can result in expression of CYP enzymes, which are associated with ROS production.[13]	
Oxidase activation (NADPH, xanthine) leading to oxidative stress	Strong	Oxidases are proteins that transport electrons across biological membranes. Upon disturbance of these proteins, ROS can be generated.[1]	
Oxidative stress leading to protein oxidation	Strong	ROS can oxidize proteins either reversibly or irreversibly.[14]	
Oxidative stress leading to lipid peroxidation	Strong	ROS can break down polyunsaturated fatty acids, damaging lipid-containing membranes.[15]	KER#1727
Oxidative stress leading to oxidative DNA damage	Strong	ROS are known to be able to interact with DNA.[16]	KER#1904
Protein oxidation leading to endoplasmic reticulum stress	Moderate	Protein oxidation can lead to misfolding of proteins, ultimately inducing the unfolded protein response and endoplasmic reticulum stress.[17]	
Protein oxidation leading to altered	Strong	Oxidation of PTEN can lead to increased cell survival through Akt signaling. Keap-	

cell survival		1 oxidation results in activation of NRF2 and subsequent expression of anti-apoptotic factors.[18-22]	
Protein oxidation leading to tumor promoting inflammation	Weak	Oxidation of IκB allows for NFκB activation, whereas oxidation of IKK prevents NFκB activation.[23-25]	
Lipid peroxidation leading to endoplasmic reticulum stress	Moderate	ROS can break down polyunsaturated fatty acids, damaging the endoplasmic reticulum membrane.[14, 26]	
Lipid peroxidation leading to altered cell survival	Strong	Mechanisms linking lipid peroxidation to cell death are well established.[27-29]	
Oxidative DNA damage leading to genomic instability	Moderate	ROS-induced DNA damage can alter the DNA methylation status and form secondary DNA structures which require special polymerases for continuation of DNA synthesis.[30, 31]	KER#1913, KER#1939
Oxidative DNA damage leading to an increase in mutations	Moderate	Oxidative DNA damage can only result in mutations if the damage is not timely repaired.	KER#1914
Oxidative DNA damage leading to inadequate DNA repair	Strong	Repair mechanisms of DNA damage are extensively studied. There is a limit to the amount of oxidative DNA damage that can be repaired.	KER#1909
Inadequate DNA repair leading to an increase in mutations	Strong	It is generally accepted that failure to (correctly) repair DNA damage results in mutations.	KER#164
Endoplasmic reticulum stress leading to altered cell survival	Strong	Disturbance of ER stress can lead to the unfolded protein response, which can eventually trigger cell death.[32]	
Altered cell survival leading to tumor promoting inflammation	Strong	Damage-associated molecular patterns (DAMPs), resulting from cell death, are known to trigger inflammation. Inflammation is a recognized risk factor for carcinogenesis.[32]	
Altered cell survival leading to sustained/regenerative proliferation	Strong	As a response to injury, cells will proliferate to replace dead cells.[33, 34]	KER#1514
Tumor promoting inflammation leading to sustained/regenerative proliferation	Weak	NFκB is an essential transcription factor for rodent liver regeneration. Better understanding of signaling pathways involved is necessary.[35-38]	
Genomic instability leading to sustained/regenerative proliferation	Strong	DNA methylation can alter the regulation of tumor-suppressor and proto-oncogenes, resulting in proliferation. Multiple studies have shown a relationship between chromosomal	KER#1979

		aberrations and increased cellular proliferation.[31, 39-41]	
Increase in mutations leading to sustained/regenerative proliferation	Strong	Mutations in either tumor-suppressor or proto-oncogenes can increase the proliferation rate in cells.	KER#1978
Sustained/regenerative proliferation leading to cancer	Strong	Diving cells are at greater risk of acquiring and maintaining a mutation. Mutations can enable malignant transformation of cells if affecting either tumor-suppressor or proto-oncogenes.	KER#1518

**B) Essentiality of key events**			
**KE**	**Level of support**	**Evidence**	**AOP wiki**
CYP activation (CYP1A, CYP1B1, CYP1D1, CYP2E1, CYP3A4)	Moderate	Cyp2e1 knockout mice do not present oxidative damage, hepatotoxicity nor regenerative proliferation following chemical exposure, whereas wildtype mice do.[3, 4]	
Mitochondrial complex inhibition	Strong	Mitochondrial complex inhibition induces mitochondrial membrane depolarization.[5]	
Mitochondrial dysfunction	Moderate	Multiple studies show that antioxidants can protect cells against rotenone-induced oxidative stress. Rotenone is a mitochondrial complex inhibitor.[42]	
Impacting cellular antioxidant capacity	Strong	Antioxidant knockout/knockdown mice were shown to be predisposed to oxidative DNA damage and carcinogenesis.[8-11]	
Sustained AhR activation	Strong	siRNA targeting AhR reduces production of superoxide and hydroxyl radicals upon chemical stimulation.[13]	
Oxidase activation (NADPH, xanthine)	Strong	ROS production was shown to be blocked by addition of a xanthine oxidase inhibitor.[43]	
Oxidative stress	Strong	Antioxidant treatment can prevent lipid peroxidation and cytotoxicity.[44]	
Protein oxidation	Moderate	Protein oxidation can induce the unfolded protein response. Oxidation of phosphateses affects signaling pathways involved in proliferation, survival and inflammation.[17, 18, 20, 23, 45-47] We are not aware of any studies specifically blocking	

		protein oxidation.	
Lipid peroxidation	Moderate	Lipid peroxidation products can activate NFκB-driven inflammation, and induce cell death.[28, 29, 48] We are not aware of any studies specifically blocking lipid peroxidation.	
Oxidative DNA damage	Strong	Modulation of cellular ROS levels causes concordant changes in the amount of oxidative DNA damage.	AOP#296, KER#1634
Endoplasmic reticulum stress	Weak	Both protein oxidation and lipid peroxidation can activate downstream key events without inducing endoplasmic reticulum stress.[29, 48] We are not aware of any studies specifically blocking endoplasmic reticulum stress.	
Altered cell survival	Weak	We are not aware of any studies specifically blocking cell death following chemical exposure.	AOP#220
Genomic instability	Moderate	Gene fusions produced by chromosomal translocations were shown to increase the proliferation rate of cells.	KER#1979
Increase in mutations	Moderate	Mutations cannot occur without untimely repair. Proliferation and mutations can hand in hand.	AOP#296, KER#185
Inadequate DNA repair	Strong	Concordant changes in mutations following disruption of DNA repair mechanisms have been demonstrated.	AOP#285, KER#155
Tumor promoting inflammation	Moderate	Rodents lacking NFκB show impaired liver regeneration. Better understanding of the exact signaling pathways involved is necessary.[35, 36]	
Sustained/regenerative proliferation	Moderate	Jnk1 knockout mice show impaired cellular proliferation and subsequent decreased tumor incidence. Better understanding of the exact signaling pathways involved is necessary.[49]	

C) Empirical evidence for adjacent key event relationships					
**KER**	**Level of support**	**Dose concordance**	**Time concordance**	**Incidence concordance**	**Contradictions**
CYP activation (CYP1A, CYP1B1, CYP1D1, CYP2E1, CYP3A4) leading to oxidative stress	Moderate	HepG2 cells overexpression CYP2E1 produce more ROS after ethanol exposure compared to HepG2 cells with normal CYP2E1 expression.[50, 51] Treatment with a CYP inhibitor decreases chemical-induced lipid peroxidation.[52]	In rats, a time-dependent increase in Cyp2E1 protein levels, superoxide radical production and lipid peroxidation was shown. AOP wiki KER#1512	An increase in CYP2E1 protein expression can be observed at concentrations which do not induce oxidative stress. [53]	Yes, it is not always clear whether oxidative stress is the result of CYP activity (electron leaking) or of a reactive metabolite.
Mitochondrial complex inhibition leading to mitochondrial dysfunction	Strong	Rotenone (a complex I inhibitor) exposure induces a dose-dependent decrease in mitochondrial membrane potential.[54]	Rotenone (a complex I inhibitor) exposure induces a time-dependent decrease in mitochondrial membrane potential.[54]	Mitochondrial complex inhibition can occur at concentrations which do not affect mitochondrial function significantly.[54]	No
Mitochondrial dysfunction leading to oxidative stress	Moderate	Rotenone (a complex I inhibitor) exposure induces a dose-dependent increase in ROS.[54] Mitochondrial complex inhibition occurs at a lower concentration compared to the concentration inducing ROS.[55]	Rotenone (a complex I inhibitor) exposure induces a time-dependent increase in ROS.[54] Mitochondrial complex inhibition precedes ROS formation.[56, 57]	Mitochondrial electron transport change inhibition was shown to be greater than the associated ROS generation.[58]	Yes, mitochondrial dysfunction can induce oxidative stress and oxidative stress can induce mitochondrial dysfunction.
Impacting cellular antioxidant capacity leading to oxidative stress	Moderate	Addition of antioxidants prevents chemical-induced lipid peroxidation.[51, 52]	No temporal evidence found.	Decreased GSH content can be found at concentrations which do not induce lipid peroxidation. [52]	Yes, a decrease in cellular antioxidants can induce oxidative stress and oxidative stress can, in turn, affect cellular antioxidant levels and activity.
Sustained AhR activation leading to oxidative stress	Moderate	AhR silencing abolishes nuclear translocation of NRF-2.[59]	AhR signaling is activated at an earlier time point following	AhR signaling is activated upon a concentration level that	Yes, it is unclear whether oxidative stress results from AhR

			ketoconazole exposure compared to NRF-2 signaling.[59]	does not induce nuclear localisation of NRF-2.[59]	activation or from activation of AhR downstream targets such as CYP1A1.
Oxidase activation (NADPH, xanthine) leading to oxidative stress	Moderate	Addition of allopurinol, a xanthine oxidase inhibitor, reduces ROS generation following azathioprine exposure .[60]	Xanthine oxidase activation results in a time-dependent increase in ROS.[43]	No information regarding incidence concordance found.	No
Oxidative stress leading to protein oxidation	Strong	A dose-dependent decrease is PTEN activity following hydrogen peroxide treatment has been observed.[20]	An increase in oxidative stress and an increase in protein oxidation are observed at similar time points following chemical exposure.[61]	An increase in protein oxidation is observed at a higher concentration compared to the concentration inducing oxidative stress.[61]	No
Oxidative stress leading to lipid peroxidation	Moderate	A decrease in ROS induces a decrease in lipid peroxidation.[62]	An increase in oxidative stress and an increase in lipid peroxidation are observed at similar time points following chemical exposure.[61]	Oxidative stress and lipid peroxidation are found at similar incidences.[61]	Yes, oxidative stress can lead to lipid peroxidation and lipid peroxidation, in turn, can propagate oxidative stress.
Oxidative stress leading to oxidative DNA damage	Strong	KER#1904	KER#1904	KER#1904	No
Protein oxidation leading to endoplasmic reticulum stress	Weak	Increased production of ROS can induce protein misfolding and oxidative stress.[17]	ROS generation and UPR target gene expression are observed at similar time points.[63]	No information regarding incidence concordance found.	Yes, oxidative stress can also induce endoplasmic reticulum stress through calcium release.[64]
Protein oxidation leading to altered cell survival	Weak	A dose-dependent decrease in PTEN activity following hydrogen peroxide treatment has been observed.[20] Chemically induced ROS induces a concentration-dependent decrease in cell viability. [65]	Chemically induced ROS precedes cell death.[65]	No information regarding incidence concordance found.	Yes, protein oxidation can both promote and reduce cell survival, dependent on the oxidized proteins.
Protein oxidation	Weak	Hydrogen peroxide	Chemically induced	No information	Yes, protein oxidation

leading to tumor promoting inflammation		dose-dependently leads to phosphorylation of IκB, allowing NFκB nuclear translocation.[23] Dose-concordance between chemically induced ROS and pro-inflammatory mediators has been observed.[66]	ROS precedes increased levels of pro-inflammatory mediators. [66]	regarding incidence concordance found.	can both stimulate and suppress inflammation depending on the level of oxidative stress and the protein oxidized.
Lipid peroxidation leading to endoplasmic reticulum stress	Weak	HNE (lipid peroxidation product) treatment was shown to increase the PERK pathway (part of the unfolded protein response).[67]	No information regarding temporal concordance found.	No information regarding incidence concordance found.	No
Lipid peroxidation leading to altered cell survival	Moderate	An increase in lipid peroxidation correlates with an increase in cell death.[52]	An increase in lipid peroxidation can be observed before an increase in cell death.[52]	Lipid peroxidation inducing concentrations precede cell death inducing concentrations (Moore 2010).	Yes, depending on the extend of lipid peroxidation cell survival can both be promoted and reduced.
Oxidative DNA damage leading to genomic instability	Moderate	AOP wiki KER#1913 & KER#1939	AOP wiki KER#1913 & KER#1939	AOP wiki KER#1913 & KER#1939	No
Oxidative DNA damage leading to an increase in mutations	Moderate	AOP wiki KER#1914	AOP wiki KER#1914	AOP wiki KER#1914	Yes, inadequate repair is essential for propagation of mutations.
Oxidative DNA damage leading to inadequate DNA repair	Moderate	AOP wiki KER#1909	AOP Wiki KER#1909	AOP wiki KER#1909	No
Inadequate DNA repair leading to an increase in mutations	Strong	AOP wiki KER#164	AOP wiki KER#164	AOP wiki KER#164	No
Endoplasmic reticulum stress leading to altered cell survival	Moderate	Deficiency in CHOP, a downstream target gene of the unfolded protein response, reduces the extent of cell death after ER stress.[68] Forced expression of CHOP increases cell death.[69]	ER stress and cell death are induced at a similar time point following chemical exposure.[69]	No information regarding incidence concordance found.	No

		Addition of an ER stress inhibitor reduces hydrogen peroxide induced cell death, whereas addition of an ER stress stimulator increases hydrogen peroxide induced cell death.[70]			
Altered cell survival leading to tumor promoting inflammation	Moderate	Hepatocyte apoptosis in mice stimulates inflammation. Upon blockage of apoptosis, the inflammatory response was inhibited.[71] Adding an apoptosis inhibitor, was shown to block the release of HMGB-1.[72]	Cell death was shown to be detectable 4 hours after chemical treatment, HMGB-1 release could be detected after 24 and 30 hours.[72]	No information regarding incidence concordance found.	Yes, cell death can lead to inflammation and inflammation, in turn, can induce cell death.
Altered cell survival leading to sustained/regenerative proliferation	Moderate	A higher induction of cell death induces a larger increase in proliferation.[73]	Chemically induced cell death occurs at an earlier time point than regenerative proliferation.[74]	Chemically induced cytotoxicity is observed at concentrations lower than those that induce regenerative proliferation.[75]	Yes, both decreased and increased cell death can result in abnormal proliferation.
Tumor promoting inflammation leading to sustained/regenerative proliferation	Weak	In a murine mammary tumor model, NFκB inhibition decreases cellular proliferation partly.[76]	Inflammation enhances compensatory proliferation.[77, 78]	No information regarding incidence concordance found.	No, but better understanding of involved signaling pathways is necessary. Differences between tissues and contexts are not well understood.
Genomic instability leading to sustained/regenerative proliferation	Moderate	AOP wiki KER#1979	AOP wiki KER#1979	AOP wiki KER#1979	Yes, genomic instability can lead to proliferation and proliferation, in turn, can lead to genomic instability.
Increase in mutations leading to sustained/regenerative	Moderate	AOP wiki KER#1978	AOP wiki KER#1978	AOP wiki KER#1978	Yes, mutations can lead to proliferation and proliferation, in turn, can

proliferation					lead to incorporation and maintenance of mutations.
Sustained/regenerative proliferation leading to cancer	Moderate	Higher BrdU labelling index correlates with tumor incidence . AOP wiki KER#1518	Proliferation can be observed within a day whereas tumor formation takes years. AOP wiki KER#1518	An increase in proliferation can be found at lower than tumor inducing concentrations. AOP wiki KER#1518	Yes, healthy regeneration can occur.

D) Overall assessment of the weight of evidence for adjacent key event relationships	
**KER**	**Evidence**
CYP activation (CYP1A, CYP1B1, CYP1D1, CYP2E1, CYP3A4) leading to oxidative stress	Moderate
Mitochondrial complex inhibition leading to mitochondrial dysfunction	Strong
Mitochondrial dysfunction leading to oxidative stress	Moderate
Impacting cellular antioxidant capacity leading to oxidative stress	Moderate
Sustained AhR activation leading to oxidative stress	Weak
Oxidase activation (NADPH, xanthine) leading to oxidative stress	Strong
Oxidative stress leading to protein oxidation	Strong
Oxidative stress leading to lipid peroxidation	Strong
Oxidative stress leading to oxidative DNA damage	Strong
Protein oxidation leading to endoplasmic reticulum stress	Moderate
Protein oxidation leading to altered cell survival	Moderate
Protein oxidation leading to tumor promoting inflammation	Weak
Lipid peroxidation leading to endoplasmic reticulum stress	Moderate
Lipid peroxidation leading to altered cell survival	Strong
Oxidative DNA damage leading to genomic instability	Moderate
Oxidative DNA damage leading to an increase in mutations	Moderate
Oxidative DNA damage leading to inadequate DNA repair	Strong
Inadequate DNA repair leading to an increase in mutations	Strong
Endoplasmic reticulum stress leading to altered cell survival	Strong
Altered cell survival leading to tumor promoting inflammation	Strong
Altered cell survival leading to sustained/regenerative proliferation	Strong
Tumor promoting inflammation leading to sustained/regenerative proliferation	Weak
Genomic instability leading to sustained/regenerative proliferation	Strong
Increase in mutations leading to sustained/regenerative proliferation	Strong
Sustained/regenerative proliferation leading to cancer	Strong

Table S2. Possible assays for the AOP network and reference chemicals for these assays.

Key event	Possible assays	Reference chemicals
CYP activation (CYP1A, CYP1B1, CYP1D1, CYP2E1, CYP3A4)	Western blot; HepRG CYP induction test[79]	Ethanol, acetaminophen, chloroform
Mitochondrial complex inhibition	Seahorse[80]	Rotenone, amiodarone
Impacting cellular antioxidant capacity	GSH:GSSG ratio[81]; antioxidant capacity[82]; antioxidant activity[83]	Diethyl maleate, pentachlorophenol, para-dichlorobenzene
Sustained AhR activation	Aryl hydrocarbon receptor transactivation assay[84]	TCDD, ketoconazole, benzo[*a*]pyrene
Oxidase activation	Dihydroethidium oxidation[85]	Nitrofurantoin, azathioprine
Mitochondrial dysfunction	TMRE mitochondrial membrane potential[86]	Rotenone, amiodarone, chlorothalonil
Oxidative stress	DCFDA[85]; NRF2 target gene expression[87]; NRF2 nuclear localization[88]; ToxTracker[89]	Diethyl maleate, tert-butyl hydroperoxide, hydrogen peroxide
Protein oxidation	Protein carbonyl content[90]	Diclofenac
Lipid peroxidation	Malondialdehyde (MDA)/thiobarbituric acid reactive substance (TBARS)[85, 91]	Carbon tetrachloride, trichloroethylene, lindane
Oxidative DNA damage	Comet (Fpg or hOGG1)[92]; 8-OHdG ELISA[93]	Cisplatin, tert-butyl hydroperoxide, arsenite
Endoplasmic reticulum stress	ToxTracker[89]	Tunicamycin, thapsigargine, cyclosporine A
Altered cell survival	MTT[94]; LDH[95]; TUNEL[96]	Carbon tetrachloride, trichloroethylene, pentachlorophenol
Genomic instability	Chromosomal abberation test (OECD TG475[97]/483[98]); DNA methylation[99]	Methapyrilene HCl, carbon tetrachloride, chloroform
Increase in mutations	Ames test (OECD TG471[100]); *hprt* mutation test (OECD TG476[101]); mouse lymphoma *tk* mutation assay (OECD TG490[102]); *in vivo* transgenic rodent (TGR) gene mutation assay (OECD TG488[103])	Cisplatin, mitomycin, aflatoxin B1
Inadequate DNA repair	Primary Rat Hepatocyte DNA Repair Assay[104]; (indirect) retention of/increase in DNA lesions (see assays 'Increase in mutations')	Radiation, ciprofloxacin

Tumor promoting inflammation	Cytokine expression[87, 88] ; ToxTracker[89]	TNFα, TGFβ1, lindane
Proliferation	Repeated dose 90-day oral toxicity study in rodents (OECD TG408[105])	Methapyrilene HCl, phenobarbital, chloroform
Cancer	Two-year carcinogenicity study in rats or mice (OECD TG451[106]/453[107])	Methapyrilene HCl, carbon tetrachloride, trichloroethylene, pentachlorophenol, lindane, chlorothalonil, arsenite

Table S3. AOPs from the AOP-wiki with oxidative stress or reactive oxygen species (ROS) as key event. Not all AOPs listed below are endorsed or open for citation yet.

#AOP	Molecular initiating event	Adverse outcome	URL
17	Binding, Thiol/seleno-proteins involved in protection against oxidative stress	Impairment, Learning and memory	https://aopwiki.org/aops/17
26	Inhibition, Ca++ ATPase	Increased, Oxidative damage	https://aopwiki.org/aops/26
27	Inhibition, Bile Salt Export Pump (ABCB11)	Cholestasis, Pathology	https://aopwiki.org/aops/27
31	N/A, Parent compound is converted to the reactive metabolite and forms free radicals leading to oxidation of heme iron(II) in hemoglobin to iron(III)	N/A, Cyanosis occurs	https://aopwiki.org/aops/31
138	Inhibition, organic anion transporter 1 (OAT1)	Increased Mortality	https://aopwiki.org/aops/138
171	X	Increased, mesotheliomas	https://aopwiki.org/aops/171
177	Inhibition, Cyclooxygenase 1 activity	Increased Mortality	https://aopwiki.org/aops/177
186	X	Increased Mortality	https://aopwiki.org/aops/186
200	Activation, Estrogen receptor	N/A, Breast Cancer	https://aopwiki.org/aops/200
207	Activation, NADPH Oxidase	Reproductive failure	https://aopwiki.org/aops/207
213	Inhibition, Fatty Acid Beta Oxidation	N/A, Steatohepatitis	https://aopwiki.org/aops/213
220	Activation of Cyp2E1	Liver Cancer	https://aopwiki.org/aops/220
260	CYP2E1 Activation	Neurodegeneration	https://aopwiki.org/aops/260
266	Decrease, Coupling of oxidative phosphorylation	Decrease, Growth	https://aopwiki.org/aops/266
267	Decrease, Coupling of oxidative phosphorylation	Decrease, Growth	https://aopwiki.org/aops/267
268	Decrease, Coupling of oxidative phosphorylation	Decrease, Growth	https://aopwiki.org/aops/268

273	Mitochondrial complex inhibition	Liver Injury	https://aopwiki.org/aops/273
284	Binding, Thiol/seleno-proteins involved in protection against oxidative stress	Chronic kidney disease	https://aopwiki.org/aops/284
293	Increase, DNA damage	N/A, Breast Cancer	https://aopwiki.org/aops/293
294	Increase in reactive oxygen and nitrogen species (RONS)	N/A, Breast Cancer	https://aopwiki.org/aops/294
298	Chronic reactive oxygen species	Treatment-resistant gastric cancer	https://aopwiki.org/aops/298
299	Deposition of Energy	Reduction, Cumulative fecundity and spawning	https://aopwiki.org/aops/299
303	Frustrated phagocytosis	Lung cancer	https://aopwiki.org/aops/303
311	Deposition of Energy	Reduction, Cumulative fecundity and spawning	https://aopwiki.org/aops/311
319	Induced dysregulation of ACE2	Lung fibrosis	https://aopwiki.org/aops/319
325	N/A, Mitochondrial dysfunction	Decrease, Fecundity	https://aopwiki.org/aops/325
326	N/A, Mitochondrial dysfunction	Decrease, Fecundity	https://aopwiki.org/aops/326
327	Increase, Reactive Oxygen Species production	Increase, Mortality	https://aopwiki.org/aops/327
328	Increase, Reactive Oxygen Species production	Increase, Mortality	https://aopwiki.org/aops/328
329	Increase, Reactive Oxygen Species production	Increase, Mortality	https://aopwiki.org/aops/329
330	Increase, Reactive Oxygen Species production	Increase, Mortality	https://aopwiki.org/aops/330
377	Prolonged TLR9 activation	Multi Organ Failure involving Acute Respiratory Distress Syndrome	https://aopwiki.org/aops/377
382	Binding of agonist, Angiotensin II receptor type 1 receptor (AT1R)	Lung fibrosis	https://aopwiki.org/aops/382
383	Induced dysregulation of ACE2	N/A, Liver fibrosis	https://aopwiki.org/aops/383

384	Hyperactivation of ACE/Ang-II/AT1R axis	Chronic kidney disease	https://aopwiki.org/aops/384
386	Deposition of Energy	Decrease, Reproduction	https://aopwiki.org/aops/386
387	Deposition of Energy	Decrease, Reproduction	https://aopwiki.org/aops/387
396	Deposition of Ionizing Energy	Decrease, Reproduction	https://aopwiki.org/aops/396
409	Frustrated phagocytosis	Increased, mesotheliomas	https://aopwiki.org/aops/409
411	Oxidative Stress	Decrease, Lung function	https://aopwiki.org/aops/411
413	Oxidation, Glutathione	Increased Mortality	https://aopwiki.org/aops/413
416	Activation, AhR	Lung cancer	https://aopwiki.org/aops/416
418	Activation, AhR	Lung cancer	https://aopwiki.org/aops/418
420	Activation, AhR	Lung cancer	https://aopwiki.org/aops/420
424	Oxidative Stress	Decrease, Lung function	https://aopwiki.org/aops/424
425	Oxidative Stress	Decrease, Lung function	https://aopwiki.org/aops/425
429	Mitochondrial dysfunction	Memory Loss	https://aopwiki.org/aops/429
437	Inhibition, Mitochondrial Electron Transport Chain Complexes	Occurrence, Kidney toxicity	https://aopwiki.org/aops/437
444	Deposition of Energy	Decrease, Reproduction	https://aopwiki.org/aops/444
447	Inhibition, Mitochondrial Electron Transport Chain Complexes	Increased, Kidney Failure	https://aopwiki.org/aops/447
450	Acetylcholinesterase (AchE) Inhibition	Increased Mortality	https://aopwiki.org/aops/450
451	Substance interaction with the lung resident cell membrane components	Lung cancer	https://aopwiki.org/aops/451
452	Non-coding RNA expression profile alteration	Chronic obstructive pulmonary disease	https://aopwiki.org/aops/452

Table S4. AOPs from the AOP-wiki with cytotoxicity as key event and cancer as adverse outcome not mentioning oxidative stress or reactive oxygen species (ROS) as key event. Not all AOPs listed below are endorsed or open for citation yet.

**#AOP**	**Molecular initiating event**	**Adverse outcome**	**URL**
32	Production, Critical Metabolites	Formation, Liver tumor	https://aopwiki.org/aops/32
41	Activation, Long term AHR receptor driven direct and indirect gene expression changes	Formation, Hepatocellular and Bile duct tumors	https://aopwiki.org/aops/41
105	Increased, Binding of chemicals to 2u (serum)	Increase, Adenomas/carcinomas (renal tubular)	https://aopwiki.org/aops/105
108	Inhibition, Pyruvate dehydrogenase kinase (PDK) enzyme	Increase, hepatocellular adenomas and carcinomas	https://aopwiki.org/aops/108
109	Increase, Cytotoxicity (club cells)	Increase, Adenomas/carcinomas (bronchioloalveolar)	https://aopwiki.org/aops/109
114	Inhibition, 4-hydroxyphenyl-pyruvate dioxygenase (HPPD) enzyme	Increase, Papillomas/carcinomas (squamous cells)	https://aopwiki.org/aops/114
115	Increase, Cytotoxicity (epithelial cells)	Increase, Papillomas/carcinomas (squamous cells)	https://aopwiki.org/aops/115
116	Increase, Cytotoxicity (tubular epithelial cells)	Increase, Adenomas/carcinomas (renal tubular)	https://aopwiki.org/aops/116
118	Increase, Cytotoxicity (hepatocytes)	Increase, hepatocellular adenomas and carcinomas	https://aopwiki.org/aops/118
121	Increase, Urinary bladder calculi	Increase, Adenomas/carcinomas (urothelial)	https://aopwiki.org/aops/121
136	Decrease, Intracellular pH	Increase, Site of Contact Nasal Tumors	https://aopwiki.org/aops/136
335	Mitochondrial impairment	Urothelial Tumor	https://aopwiki.org/aops/335

REFERENCES

(1) Hrycay, E. G.; Bandiera, S. M. Involvement of Cytochrome P450 in Reactive Oxygen Species Formation and Cancer. *Adv Pharmacol* **2015**, *74*, 35-84. DOI: 10.1016/bs.apha.2015.03.003.
(2) Veith, A.; Moorthy, B. Role of cytochrome P450s in the generation and metabolism of reactive oxygen species. *Curr Opin Toxicol* **2018**, *7*, 44-51. DOI: 10.1016/j.cotox.2017.10.003.
(3) Constan, A. A.; Sprankle, C. S.; Peters, J. M.; Kedderis, G. L.; Everitt, J. I.; Wong, B. A.; Gonzalez, F. L.; Butterworth, B. E. Metabolism of Chloroform by Cytochrome P450 2E1 Is Required for Induction of Toxicity in the Liver, Kidney, and Nose of Male Mice. *Toxicol Appl Pharmacol* **1999**, *160* (2), 120-126. DOI: 10.1006/taap.1999.8756.
(4) Bradford, B. U.; Kono, H.; Isayama, F.; Kosyk, O.; Wheeler, M. D.; Akiyama, T. E.; Bleye, L.; Krausz, K. W.; Gonzalez, F. J.; Koop, D. R.; et al. Cytochrome P450 CYP2E1, but not nicotinamide adenine dinucleotide phosphate oxidase, is required for ethanol-induced oxidative DNA damage in rodent liver. *Hepatology* **2005**, *41* (2), 336-344. DOI: 10.1002/hep.20532.
(5) Katila, N.; Bhurtel, S.; Park, P. H.; Choi, D. Y. Metformin attenuates rotenone-induced oxidative stress and mitochondrial damage via the AKT/Nrf2 pathway. *Neurochem Int* **2021**, *148* (1872-9754 (Electronic)). DOI: 10.1016/j.neuint.2021.105120.
(6) Yang, C.; Lim, W.; Song, G. Mediation of oxidative stress toxicity induced by pyrethroid pesticides in fish. *Comp Biochem Physiol C Toxicol Pharmacol* **2020**, *234*. DOI: 10.1016/j.cbpc.2020.108758.
(7) Patlevic, P.; Vaskova, J.; Svorc, P., Jr.; Vasko, L.; Svorc, P. Reactive oxygen species and antioxidant defense in human gastrointestinal diseases. *Integr Med Res* **2016**, *5* (4), 250-258. DOI: 10.1016/j.imr.2016.07.004.
(8) Busuttil, R. A.; Garcia, A. M.; Cabrera, C.; Rodriguez, A.; Suh, Y.; Kim, W. H.; Huang, T.-T.; Vijg, J. Organ-Specific Increase in Mutation Accumulation and Apoptosis Rate in CuZn-Superoxide Dismutase–Deficient Mice. *Cancer Res* **2005**, *65* (24), 11271-11275. DOI: 10.1158/0008-5472.Can-05-2980 (acccessed 2022-02-06).
(9) Elchuri, S.; Oberley, T. D.; Qi, W.; Eisenstein, R. S.; Jackson Roberts, L.; Van Remmen, H.; Epstein, C. J.; Huang, T.-T. CuZnSOD deficiency leads to persistent and widespread oxidative damage and hepatocarcinogenesis later in life. *Oncogene* **2005**, *24* (3), 367-380. DOI: 10.1038/sj.onc.1208207.
(10) Neumann, C. A.; Krause, D. S.; Carman, C. V.; Das, S.; Dubey, D. P.; Abraham, J. L.; Bronson, R. T.; Fujiwara, Y.; Orkin, S. H.; Van Etten, R. A. Essential role for the peroxiredoxin Prdx1 in erythrocyte antioxidant defence and tumor suppression. *Nature* **2003**, *424* (6948), 561-565. DOI: 10.1038/nature01819.
(11) Rani, V.; Neumann, C. A.; Shao, C.; Tischfield, J. A. Prdx1 deficiency in mice promotes tissue specific loss of heterozygosity mediated by deficiency in DNA repair and increased oxidative stress. *Mutat Res* **2012**, *735* (1), 39-45. DOI: 10.1016/j.mrfmmm.2012.04.004.
(12) Chu, F. F.; Esworthy, R. S.; Chu, P. G.; Longmate, J. A.; Huycke, M. M.; Wilczynski, S.; Doroshow, J. H. Bacteria-Induced Intestinal Cancer in Mice with Disrupted Gpx1 and Gpx2 Genes. *Cancer Res* **2004**, *64* (3), 962-968. DOI: 10.1158/0008-5472.Can-03-2272.
(13) Kopf, P. G.; Walker, M. K. 2,3,7,8-Tetrachlorodibenzo-p-dioxin increases reactive oxygen species production in human endothelial cells via induction of cytochrome P4501A1. *Toxicol Appl Pharmacol* **2010**, *245* (1), 91-99. DOI: 10.1016/j.taap.2010.02.007.
(14) Jabłońska-Trypuć, A. Pesticides as Inducers of Oxidative Stress. *React Oxyg Species* **2017**, *3* (8), 96-110. DOI: 10.20455/ros.2017.823.
(15) Hauck, A. K.; Bernlohr, D. A. Oxidative stress and lipotoxicity. *J Lipid Res* **2016**, *57* (11), 1976-1986. DOI: 10.1194/jlr.R066597.
(16) Kumari, S.; Badana, A. K.; G, M. M.; G, S.; Malla, R. Reactive Oxygen Species: A Key Constituent in Cancer Survival. *Biomark Insights* **2018**, *13*. DOI: 10.1177/1177271918755391.
(17) Chen, A. C. H.; Burr, L.; McGuckin, M. A. Oxidative and endoplasmic reticulum stress in respiratory disease. *Clin Transl Immunology* **2018**, *7* (6). DOI: 10.1002/cti2.1019.
(18) Lee, S. R.; Yang, K. S.; Kwon, J.; Lee, C.; Jeong, W.; Rhee, S. G. Reversible Inactivation of the Tumor Suppressor PTEN by H2O2. *J Biol Chem* **2002**, *277* (23), 20336-20342. DOI: 10.1113/expphysiol.1997.sp004024.
(19) Moloney, J. N.; Cotter, T. G. ROS signaling in the biology of cancer. *Semin Cell Dev Biol* **2018**, *80*, 50-64. DOI: 10.1016/j.semcdb.2017.05.023.
(20) Leslie, N. R.; Bennett, D.; Lindsay, Y. E.; Stewart, H.; Gray, A.; Downes, C. P. Redox regulation of PI 3-kinase signaling via inactivation of PTEN. *EMBO Journal* **2003**, *22* (20), 5501-5510. DOI: 10.1093/emboj/cdg513.

(21) Niture, S. K.; Jaiswal, A. K. Nrf2-induced antiapoptotic Bcl-xL protein enhances cell survival and drug resistance. *Free Radic Biol Med* **2013**, *57*, 119-131. DOI: 10.1016/j.freeradbiomed.2012.12.014.
(22) Niture, S. K.; Jaiswal, A. K. Nrf2 Protein Up-regulates Antiapoptotic Protein Bcl-2 and Prevents Cellular Apoptosis. *J Biol Chem* **2012**, *287* (13), 9873-9886. DOI: 10.1074/jbc.M111.312694.
(23) Zhang, J.; Johnston G Fau - Stebler, B.; Stebler B Fau - Keller, E. T.; Keller, E. T. Hydrogen peroxide activates NFkappaB and the interleukin-6 promoter through NFkappaB-inducing kinase. *Antioxid Redox Signal* **2001**, *3* (3), 493-504. DOI: 10.1089/15230860152409121.
(24) Korn, S. H.; Wouters, E. F.; Vos, N.; Janssen-Heininger, Y. M. Cytokine-induced activation of nuclear factor-kappa B is inhibited by hydrogen peroxide through oxidative inactivation of IkappaB kinase. *J Biol Chem* **2001**, *376* (38), 35693-35700. DOI: 10.1074/jbc.M104321200.
(25) Reynaert, N. L.; van der Vliet, A.; Guala, A. S.; McGovern, T.; Hristova, M.; Pantano, C.; Heintz, N. H.; Heim, J.; Ho, Y. S.; Matthews, D. E.; et al. Dynamic redox control of NF-kappaB through glutaredoxin-regulated S-glutathionylation of inhibitory kappaB kinase beta. *Proc Natl Acad Sci USA* **2006**, *103* (35), 13086-13091. DOI: 10.1073/pnas.0603290103.
(26) Kaweme, N. M.; Zhou, S.; Changwe, G. J.; Zhou, F. The significant role of redox system in myeloid leukemia: from pathogenesis to therapeutic applications. *Biomark Res* **2020**, *8* (1), Review. DOI: 10.1186/s40364-020-00242-z.
(27) Vatsyayan, R. C., P. Sharma, A.; Sharma, R.; Rao Lelsani, P. C. A., S.; Awasthi, Y. C. Role of 4-hydroxynonenal in epidermal growth factor receptor-mediated signaling in retinal pigment epithelial cells. *Exp Eye Res* **2011**, *92* (2). DOI: 10.1016/j.exer.2010.11.010.
(28) Bartsch, H.; Nair, J. Oxidative stress and lipid peroxidation-derived DNA-lesions in inflammation driven carcinogenesis. *Cancer Detect Prev* **2004**, *28* (6). DOI: 10.1016/j.cdp.2004.07.004.
(29) Yadav, U. C.; Ramana, K. V. Regulation of NF-kappaB-induced inflammatory signaling by lipid peroxidation-derived aldehydes. *Oxid Med Cell Longev* **2013**, *2013*. DOI: 10.1155/2013/690545.
(30) Stein, M.; Eckert, K. A. Impact of G-Quadruplexes and Chronic Inflammation on Genome Instability: Additive Effects during Carcinogenesis. *Genes* **2021**, *12* (11). DOI: 10.3390/genes12111779.
(31) Nishida, N.; Arizumi, T.; Takita, M.; Kitai, S.; Yada, N.; Hagiwara, S.; Inoue, T.; Minami, Y.; Ueshima, K.; Sakurai, T.; et al. Reactive oxygen species induce epigenetic instability through the formation of 8-hydroxydeoxyguanosine in human hepatocarcinogenesis. *Dig Dis* **2013**, *31* (5-6), 459-466. DOI: 10.1159/000355245.
(32) Brenner, C.; Galluzzi, L.; Kepp, O.; Kroemer, G. Decoding cell death signals in liver inflammation. *J Hepatol* **2013**, *59* (3), 583-594. DOI: 10.1016/j.jhep.2013.03.033.
(33) Luedde, T.; Kaplowitz, N.; Schwabe, R. F. Cell death and cell death responses in liver disease: mechanisms and clinical relevance. *Gastroenterology* **2014**, *147* (4), 765-783. DOI: 10.1053/j.gastro.2014.07.018.
(34) Boobis, A. R.; Daston, G. P.; Preston, R. J.; Olin, S. S. Application of key events analysis to chemical carcinogens and noncarcinogens. *Crit Rev Food Sci Nutr* **2009**, *49* (8), 690-707. DOI: 10.1080/10408390903098673.
(35) Behrens, A.; Sibilia, M.; David, J. P.; Möhle-Steinlein, U.; Tronche, F.; Schütz, G.; Wagner, E. F. Impaired postnatal hepatocyte proliferation and liver regeneration in mice lacking. *EMBO J.* **2002**, *21*, 1782-1790. DOI: 10.1093/emboj/21.7.1782.
(36) Schrum, L. W.; Black, D.; Iimuro, Y.; Rippe, R. A.; Brenner, D. A.; Behrns, K. E. c-Jun Does Not Mediate Hepatocyte Apoptosis Following NFκB Inhibition and Partial Hepatectomy. *J Surg Res* **2000**, *88* (2), 142-149. DOI: 10.1006/jsre.1999.5784.
(37) Maeda, S.; Kamata, H.; Luo, J. L.; Leffert, H.; Karin, M. IKKbeta couples hepatocyte death to cytokine-driven compensatory proliferation that promotes chemical hepatocarcinogenesis. *Cell* **2005**, *121* (7), 977-990. DOI: 10.1016/j.cell.2005.04.014.
(38) Sakurai, T.; He, G.; Matsuzawa, A.; Yu, G.; Maeda, S.; Hardiman, G.; Karin, M. Hepatocyte Necrosis Induced by Oxidative Stress and IL-1α Release Mediate Carcinogen-Induced Compensatory Proliferation and Liver Tumorigenesis. *Cancer Cell* **2008**, *14* (2), 156-165. DOI: 10.1016/j.ccr.2008.06.016.
(39) Kuppusamy, S. P.; Kaiser, J. P.; Wesselkamper, S. C. Epigenetic Regulation in Environmental Chemical Carcinogenesis and its Applicability in Human Health Risk Assessment. *Int J Toxicol* **2015**, *34* (5), 384-392. DOI: 10.1177/1091581815599350.
(40) Stefanska, B.; Huang, J.; Bhattacharyya, B.; Suderman, M.; Hallett, M.; Han, Z. G.; Szyf, M. Definition of the landscape of promoter DNA hypomethylation in liver cancer. *Cancer Res* **2011**, *71* (17), 5891-5903. DOI: 10.1158/0008-5472.CAN-10-3823.

(41) Fleisher, A. S.; Esteller, M.; Harpaz, N.; Leytin, A.; Rashid, A.; Xu, Y.; Liang, J.; Stine, O. C.; Yin, J.; Zou, T.-T.; et al. Microsatellite Instability in Inflammatory Bowel Disease-associated Neoplastic Lesions Is Associated with Hypermethylation and Diminished Expression of the DNA Mismatch Repair Gene, hMLH11. *Cancer Res* **2000**, *60* (17), 4864-4868.
(42) Saravanan, K. S.; Sindhu, K. M.; Senthilkumar, K. S.; Mohanakumar, K. P. L-deprenyl protects against rotenone-induced, oxidative stress-mediated dopaminergic neurodegeneration in rats. *Neurochem Int* **2006**, *49* (1), 28-40. DOI: 10.1016/j.neuint.2005.12.016.
(43) Abramov, A. Y.; Scorziello, A.; Duchen, M. R. Three distinct mechanisms generate oxygen free radicals in neurons and contribute to cell death during anoxia and reoxygenation. *J Neurosci* **2007**, *27* (5), 1129-1138. DOI: 10.1523/JNEUROSCI.4468-06.2007.
(44) Beddowes, E. J.; Faux, S. P.; Chipman, J. K. Chloroform, carbon tetrachloride and glutathione depletion induce secondary genotoxicity in liver cells via oxidative stress. *Toxicology* **2003**, *187* (2-3), 101-115. DOI: 10.1016/s0300-483x(03)00058-1.
(45) Chan, D. W.; Liu, V. W. S.; Tsao, G. S. W.; Yao, K.-M.; Furukawa, T.; Chan, K. K. L.; Ngan, H. Y. S. Loss of MKP3 mediated by oxidative stress enhances tumorigenicity and chemoresistance of ovarian cancer cells. *Carcinogenesis* **2008**, *29* (9), 1742-1750. DOI: 10.1093/carcin/bgn167.
(46) Salmeen, A.; Andersen, J. N.; Myers, M. P.; Meng, T. C.; Hinks, J. A.; Tonks, N. K.; Barford, D. Redox regulation of protein tyrosine phosphatase 1B involves a sulphenyl-amide intermediate. *Nature* **2003**, *423* (6941), 769-773, Article. DOI: 10.1038/nature01680.
(47) Seth, D.; Rudolph, J. Redox Regulation of MAP Kinase Phosphatase 3. *Biochemistry* **2006**, *45* (28), 8476-8487. DOI: 10.1021/bi060157p.
(48) Lei, L.; Yang, J.; Zhang, J.; Zhang, G. The lipid peroxidation product EKODE exacerbates colonic inflammation and colon tumorigenesis. *Redox Biol* **2021**, *42*, 101880. DOI: /10.1016/j.redox.2021.101880.
(49) Hui, L.; Zatloukal, K.; Scheuch, H.; Stepniak, E.; Wagner, E. F. Proliferation of human HCC cells and chemically induced mouse liver cancers requires JNK1-dependent p21 downregulation. *J Clin Invest* **2008**, *118* (12), 3943-3953. DOI: 10.1172/JCI37156.
(50) Bardag-Gorce, F.; French, B. A.; Nan, L.; Song, H.; Nguyen, S. K.; Yong, H.; Dede, J.; French, S. W. CYP2E1 induced by ethanol causes oxidative stress, proteasome inhibition and cytokeratin aggresome (Mallory body-like) formation. *Exp Mol Pathol* **2006**, *81* (3), 191-201. DOI: 10.1016/j.yexmp.2006.07.007.
(51) Wu, D.; Cederbaum, A. I. Oxidative stress mediated toxicity exerted by ethanol-inducible CYP2E1. *Toxicol Appl Pharmacol* **2005**, *207* (2 Suppl), 70-76. DOI: 10.1016/j.taap.2005.01.057.
(52) Suzuki, T.; Nojiri, H.; Isono, H.; Ochi, T. Oxidative damages in isolated rat hepatocytes treated with the organochlorine fungicides captan, dichlofluanid and chlorothalonil. *Toxicology* **2004**, *204* (2-3), 97-107. DOI: 10.1016/j.tox.2004.06.025.
(53) Valencia-Olvera, A. C.; Moran, J.; Camacho-Carranza, R.; Prospero-Garcia, O.; Espinosa-Aguirre, J. J. CYP2E1 induction leads to oxidative stress and cytotoxicity in glutathione-depleted cerebellar granule neurons. *Toxicol In Vitro* **2014**, *28* (7), 1206-1214. DOI: 10.1016/j.tiv.2014.05.014.
(54) Barrientos, A.; Moraes, C. T. Titrating the effects of mitochondrial complex I impairment in the cell physiology. *J Biol Chem* **1999**, *274* (23), 16188-16197. DOI: 10.1074/jbc.274.23.16188.
(55) Miyayama, T.; Arai, Y.; Suzuki, N.; Hirano, S. Mitochondrial electron transport is inhibited by disappearance of metallothionein in human bronchial epithelial cells following exposure to silver nitrate. *Toxicology* **2013**, *305*, 20-29. DOI: 10.1016/j.tox.2013.01.004.
(56) Kruidering, M.; Van de Water, B.; de Heer, E.; Mulder, G. J.; Nagelkerke, J. F. Cisplatin-induced nephrotoxicity in porcine proximal tubular cells: mitochondrial dysfunction by inhibition of complexes I to IV of the respiratory chain. *J Pharmacol Exp Ther* **1997**, *280* (2), 638-649.
(57) Garcia-Nino, W. R.; Tapia, E.; Zazueta, C.; Zatarain-Barron, Z. L.; Hernandez-Pando, R.; Vega-Garcia, C. C.; Pedraza-Chaverri, J. Curcumin pretreatment prevents potassium dichromate-induced hepatotoxicity, oxidative stress, decreased respiratory complex I activity, and membrane permeability transition pore opening. *Evid Based Complement Alternat Med* **2013**, *2013*, 424692. DOI: 10.1155/2013/424692.
(58) Prakash, C.; Soni, M.; Kumar, V. Biochemical and Molecular Alterations Following Arsenic-Induced Oxidative Stress and Mitochondrial Dysfunction in Rat Brain. *Biol Trace Elem Res* **2015**, *167* (1), 121-129. DOI: 10.1007/s12011-015-0284-9.
(59) Tsuji, G.; Takahara, M.; Uchi, H.; Matsuda, T.; Chiba, T.; Takeuchi, S.; Yasukawa, F.; Moroi, Y.; Furue, M. Identification of ketoconazole as an AhR-Nrf2 activator in cultured human keratinocytes: the basis of its anti-inflammatory effect. *J Invest Dermatol* **2012**, *132* (1), 59-68. DOI: 10.1038/jid.2011.194.

(60) Al Maruf, A.; Wan, L.; O'Brien, P. J. Evaluation of azathioprine-induced cytotoxicity in an in vitro rat hepatocyte system. *Biomed Res Int* **2014**, *2014*, 379748. DOI: 10.1155/2014/379748.
(61) Li, Z. H.; Zlabek, V.; Grabic, R.; Li, P.; Machova, J.; Velisek, J.; Randak, T. Effects of exposure to sublethal propiconazole on the antioxidant defense system and Na+-K+-ATPase activity in brain of rainbow trout, Oncorhynchus mykiss. *Aquat Toxicol* **2010**, *98* (3), 297-303. DOI: 10.1016/j.aquatox.2010.02.017.
(62) Nosal, R.; Drabikova, K.; Jancinova, V.; Perecko, T.; Ambrozova, G.; Ciz, M.; Lojek, A.; Pekarova, M.; Smidrkal, J.; Harmatha, J. On the molecular pharmacology of resveratrol on oxidative burst inhibition in professional phagocytes. *Oxid Med Cell Longev* **2014**, *2014*, 706269. DOI: 10.1155/2014/706269.
(63) Tsedensodnom, O.; Vacaru, A. M.; Howarth, D. L.; Yin, C.; Sadler, K. C. Ethanol metabolism and oxidative stress are required for unfolded protein response activation and steatosis in zebrafish with alcoholic liver disease. *Dis Model Mech* **2013**, *6* (5), 1213-1226. DOI: 10.1242/dmm.012195.
(64) Farrukh, M. R.; Nissar, U. A.; Afnan, Q.; Rafiq, R. A.; Sharma, L.; Amin, S.; Kaiser, P.; Sharma, P. R.; Tasduq, S. A. Oxidative stress mediated Ca(2+) release manifests endoplasmic reticulum stress leading to unfolded protein response in UV-B irradiated human skin cells. *J Dermatol Sci* **2014**, *75* (1), 24-35. DOI: 10.1016/j.jdermsci.2014.03.005.
(65) Lu, T. H.; Hsieh, S. Y.; Yen, C. C.; Wu, H. C.; Chen, K. L.; Hung, D. Z.; Chen, C. H.; Wu, C. C.; Su, Y. C.; Chen, Y. W.; et al. Involvement of oxidative stress-mediated ERK1/2 and p38 activation regulated mitochondria-dependent apoptotic signals in methylmercury-induced neuronal cell injury. *Toxicol Lett* **2011**, *204* (1), 71-80. DOI: 10.1016/j.toxlet.2011.04.013.
(66) Chen, B.; Zhang, Y. S.; Li, G.; Cho, J. L.; Deng, Y. L.; Li, Y. J. The Impacts of Simulated Microgravity on Rat Brain Depended on Durations and Regions. *Biomed Environ Sci* **2019**, *32* (7), 496-507. DOI: 10.3967/bes2019.067.
(67) Haberzettl, P.; Hill, B. G. Oxidized lipids activate autophagy in a JNK-dependent manner by stimulating the endoplasmic reticulum stress response. *Redox Biol* **2013**, *1* (1), 56-64. DOI: 10.1016/j.redox.2012.10.003.
(68) Tamaki, N.; Hatano, E.; Taura, K.; Tada, M.; Kodama, Y.; Nitta, T.; Iwaisako, K.; Seo, S.; Nakajima, A.; Ikai, I.; et al. CHOP deficiency attenuates cholestasis-induced liver fibrosis by reduction of hepatocyte injury. *Am J Physiol Gastrointest Liver Physiol* **2008**, *294* (2), G498-505. DOI: 10.1152/ajpgi.00482.2007.
(69) Uzi, D.; Barda, L.; Scaiewicz, V.; Mills, M.; Mueller, T.; Gonzalez-Rodriguez, A.; Valverde, A. M.; Iwawaki, T.; Nahmias, Y.; Xavier, R.; et al. CHOP is a critical regulator of acetaminophen-induced hepatotoxicity. *J Hepatol* **2013**, *59* (3), 495-503. DOI: 10.1016/j.jhep.2013.04.024.
(70) Wu, Z.; Wang, H.; Fang, S.; Xu, C. Roles of endoplasmic reticulum stress and autophagy on H2O2-induced oxidative stress injury in HepG2 cells. *Mol Med Rep* **2018**, *18* (5), 4163-4174. DOI: 10.3892/mmr.2018.9443.
(71) Faouzi, S.; Burckhardt, B. E.; Hanson, J. C.; Campe, C. B.; Schrum, L. W.; Rippe, R. A.; Maher, J. J. Anti-Fas induces hepatic chemokines and promotes inflammation by an NF-kappa B-independent, caspase-3-dependent pathway. *J Biol Chem* **2001**, *276* (52), 49077-49082. DOI: 10.1074/jbc.M109791200.
(72) Bell, C. W.; Jiang, W.; Reich, C. F., 3rd; Pisetsky, D. S. The extracellular release of HMGB1 during apoptotic cell death. *Am J Physiol Cell Physiol* **2006**, *291* (6), C1318-1325. DOI: 10.1152/ajpcell.00616.2005.
(73) Ding, W.; Petibone, D. M.; Latendresse, J. R.; Pearce, M. G.; Muskhelishvili, L.; White, G. A.; Chang, C. W.; Mittelstaedt, R. A.; Shaddock, J. G.; McDaniel, L. P.; et al. In vivo genotoxicity of furan in F344 rats at cancer bioassay doses. *Toxicol Appl Pharmacol* **2012**, *261* (2), 164-171. DOI: 10.1016/j.taap.2012.03.021.
(74) Hickling, K. C.; Hitchcock, J. M.; Oreffo, V.; Mally, A.; Hammond, T. G.; Evans, J. G.; Chipman, J. K. Evidence of oxidative stress and associated DNA damage, increased proliferative drive, and altered gene expression in rat liver produced by the cholangiocarcinogenic agent furan. *Toxicol Pathol* **2010**, *38* (2), 230-243. DOI: 10.1177/0192623309357946.
(75) Moser, G. J.; Foley, J.; Burnett, M.; Goldsworthy, T. L.; Maronpot, R. Furan-induced dose-response relationships for liver cytotoxicity, cell proliferation, and tumorigenicity (furan-induced liver tumorigenicity). *Exp Toxicol Pathol* **2009**, *61* (2), 101-111. DOI: 10.1016/j.etp.2008.06.006.
(76) Connelly, L.; Barham, W.; Onishko, H. M.; Sherrill, T.; Chodosh, L. A.; Blackwell, T. S.; Yull, F. E. Inhibition of NF-kappa B activity in mammary epithelium increases tumor latency and decreases tumor burden. *Oncogene* **2011**, *30* (12), 1402-1412. DOI: 10.1038/onc.2010.521.
(77) Landen, N. X.; Li, D.; Stahle, M. Transition from inflammation to proliferation: a critical step during wound healing. *Cell Mol Life Sci* **2016**, *73* (20), 3861-3885. DOI: 10.1007/s00018-016-2268-0.
(78) Kiraly, O.; Gong, G.; Olipitz, W.; Muthupalani, S.; Engelward, B. P. Inflammation-induced cell proliferation potentiates DNA damage-induced mutations in vivo. *PLoS Genet* **2015**, *11* (2), e1004901. DOI: 10.1371/journal.pgen.1004901.
(79) Bernasconi, C.; Pelkonen, O.; Andersson, T. B.; Strickland, J.; Wilk-Zasadna, I.; Asturiol, D.; Cole, T.; Liska, R.; Worth, A.; Muller-Vieira, U.; et al. Validation of in vitro methods for human cytochrome P450 enzyme

induction: Outcome of a multi-laboratory study. *Toxicol In Vitro* **2019**, *60*, 212-228. DOI: 10.1016/j.tiv.2019.05.019.
(80) Leung, D. T. H.; Chu, S. Measurement of Oxidative Stress: Mitochondrial Function Using the Seahorse System. *Methods Mol Biol* **2018**, *1710*, 285-293. DOI: 10.1007/978-1-4939-7498-6_22.
(81) Biesemann, N.; Ried, J. S.; Ding-Pfennigdorff, D.; Dietrich, A.; Rudolph, C.; Hahn, S.; Hennerici, W.; Asbrand, C.; Leeuw, T.; Strubing, C. High throughput screening of mitochondrial bioenergetics in human differentiated myotubes identifies novel enhancers of muscle performance in aged mice. *Sci Rep* **2018**, *8* (1), 9408. DOI: 10.1038/s41598-018-27614-8.
(82) Ansermet, C.; Centeno, G.; Pradervand, S.; Harmacek, D.; Garcia, A.; Daraspe, J.; Kocherlakota, S.; Baes, M.; Bignon, Y.; Firsov, D. Renal tubular peroxisomes are dispensable for normal kidney function. *JCI Insight* **2022**, *7* (4). DOI: 10.1172/jci.insight.155836.
(83) Gong, F.; Peng, X.; Sang, Y.; Qiu, M.; Luo, C.; He, Z.; Zhao, X.; Tong, A. Dichloroacetate induces protective autophagy in LoVo cells: involvement of cathepsin D/thioredoxin-like protein 1 and Akt-mTOR-mediated signaling. *Cell Death Dis* **2013**, *4* (11), e913. DOI: 10.1038/cddis.2013.438.
(84) Han, D.; Nagy, S. R.; Denison, M. S. Comparison of recombinant cell bioassays for the detection of Ah receptor agonists. *Biofactors* **2004**, *20* (1), 11-22. DOI: 10.1002/biof.5520200102.
(85) Griendling, K. K.; Touyz, R. M.; Zweier, J. L.; Dikalov, S.; Chilian, W.; Chen, Y. R.; Harrison, D. G.; Bhatnagar, A.; American Heart Association Council on Basic Cardiovascular, S. Measurement of Reactive Oxygen Species, Reactive Nitrogen Species, and Redox-Dependent Signaling in the Cardiovascular System: A Scientific Statement From the American Heart Association. *Circ Res* **2016**, *119* (5), e39-75. DOI: 10.1161/RES.0000000000000110.
(86) Li, X.; Kang, B.; Eom, Y.; Zhong, J.; Lee, H. K.; Kim, H. M.; Song, J. S. Comparison of cytotoxicity effects induced by four different types of nanoparticles in human corneal and conjunctival epithelial cells. *Sci Rep* **2022**, *12* (1), 155. DOI: 10.1038/s41598-021-04199-3.
(87) Forlenza, M.; Kaiser, T.; Savelkoul, H. F.; Wiegertjes, G. F. The use of real-time quantitative PCR for the analysis of cytokine mRNA levels. *Methods Mol Biol* **2012**, *820*, 7-23. DOI: 10.1007/978-1-61779-439-1_2.
(88) Amsen, D.; de Visser, K. E.; Town, T. Approaches to determine expression of inflammatory cytokines. *Methods Mol Biol* **2009**, *511*, 107-142. DOI: 10.1007/978-1-59745-447-6_5.
(89) Hendriks, G.; Derr, R. S.; Misovic, B.; Morolli, B.; Calleja, F. M.; Vrieling, H. The Extended ToxTracker Assay Discriminates Between Induction of DNA Damage, Oxidative Stress, and Protein Misfolding. *Toxicol Sci* **2016**, *150* (1), 190-203. DOI: 10.1093/toxsci/kfv323.
(90) Ajima, M. N. O.; Kumar, K.; Poojary, N.; Pandey, P. K. Sublethal diclofenac induced oxidative stress, neurotoxicity, molecular responses and alters energy metabolism proteins in Nile tilapia, Oreochromis niloticus. *Environ Sci Pollut Res Int* **2021**, *28* (32), 44494-44504. DOI: 10.1007/s11356-021-13899-2 .
(91) Ping, Z.; Peng, Y.; Lang, H.; Xinyong, C.; Zhiyi, Z.; Xiaocheng, W.; Hong, Z.; Liang, S. Oxidative Stress in Radiation-Induced Cardiotoxicity. *Oxid Med Cell Longev* **2020**, *2020*, 3579143. DOI: 10.1155/2020/3579143.
(92) Smith, C. C.; O'Donovan, M. R.; Martin, E. A. hOGG1 recognizes oxidative damage using the comet assay with greater specificity than FPG or ENDOIII. *Mutagenesis* **2006**, *21* (3), 185-190. DOI: 10.1093/mutage/gel019.
(93) Breton, J.; Sichel, F.; Bianchini, F.; Prévost, V. Measurement of 8-Hydroxy-2′-Deoxyguanosine by a Commercially Available ELISA Test: Comparison with HPLC/Electrochemical Detection in Calf Thymus DNA and Determination in Human Serum. *Analytical Letters* **2003**, *36*, 123 - 134.
(94) Riss, T. L.; Moravec, R. A.; Niles, A. L.; Duellman, S.; Benink, H. A.; Worzella, T. J.; Minor, L. Cell Viability Assays. In *Assay Guidance Manual*, Markossian, S., Grossman, A., Brimacombe, K., Arkin, M., Auld, D., Austin, C., Baell, J., Chung, T. D. Y., Coussens, N. P., Dahlin, J. L., et al. Eds.; 2004.
(95) Chan, F. K.; Moriwaki, K.; De Rosa, M. J. Detection of necrosis by release of lactate dehydrogenase activity. *Methods Mol Biol* **2013**, *979*, 65-70. DOI: 10.1007/978-1-62703-290-2_7.
(96) Lozano, G. M.; Bejarano, I.; Espino, J.; Gonzalez, D.; Ortiz, A.; Garcia, J. F.; Rodriguez, A. B.; Pariente, J. A. Relationship between caspase activity and apoptotic markers in human sperm in response to hydrogen peroxide and progesterone. *J Reprod Dev* **2009**, *55* (6), 615-621. DOI: 10.1262/jrd.20250.
(97) OECD. *Test No. 475: Mammalian Bone Marrow Chromosomal Aberration Test*; 2016. DOI: doi: 10.1787/9789264264786-en (accessed 2023-03-09).
(98) OECD. *Test No. 483: Mammalian Spermatogonial Chromosomal Aberration Test*; 2016. DOI: doi:10.1787/9789264264847-en (accessed 2023-03-09).
(99) Greally, J. M.; Jacobs, M. N. In vitro and in vivo testing methods of epigenomic endpoints for evaluating endocrine disruptors. *ALTEX* **2013**, *30* (4), 445-471. DOI: 10.14573/altex.2013.4.445.

(100) OECD. *Test No. 471: Bacterial Reverse Mutation Test*; 2020. DOI: doi:10.1787/9789264071247-en (accessed 2023-03-09).
(101) OECD. *Test No. 476: In Vitro Mammalian Cell Gene Mutation Tests using the Hprt and xprt genes*; 2016. DOI: doi:10.1787/9789264264809-en (accessed 2023-03-09).
(102) OECD. *Test No. 490: In Vitro Mammalian Cell Gene Mutation Tests Using the Thymidine Kinase Gene*; 2016. DOI: doi:10.1787/9789264264908-en (accessed 2023-03-09).
(103) OECD. *Test No. 488: Transgenic Rodent Somatic and Germ Cell Gene Mutation Assays*; 2022. DOI: doi:10.1787/9789264203907-en (accessed 2023-03-09).
(104) Jeffrey, A. M.; Williams, G. M. Lack of DNA-damaging activity of five non-nutritive sweeteners in the rat hepatocyte/DNA repair assay. *Food Chem Toxicol* **2000**, *38* (4), 335-338. DOI: 10.1016/s0278-6915(99)00163-5.
(105) Wood, C. E.; Hukkanen, R. R.; Sura, R.; Jacobson-Kram, D.; Nolte, T.; Odin, M.; Cohen, S. M. Scientific and Regulatory Policy Committee (SRPC) Review: Interpretation and Use of Cell Proliferation Data in Cancer Risk Assessment. *Toxicol Pathol* **2015**, *43* (6), 760-775. DOI: 10.1177/0192623315576005.
(106) OECD. *Test No. 451: Carcinogenicity Studies*; Paris, 2018. DOI: 10.1787/9789264071186-en (accessed 2023-03-09).
(107) OECD. *Test No. 453: Combined Chronic Toxicity/Carcinogenicity Studies*; 2018. DOI: doi:10.1787/9789264071223-en (accessed 2023-03-09).
